# Inflammation Cascade-Directed Therapy by Biomimetic Polydopamine Nanosystem for Long-Term Management of Ischemic Stroke

**DOI:** 10.7150/thno.118196

**Published:** 2026-01-01

**Authors:** Yuyi Zheng, Xiaojie Chen, Qi Zhang, Ziwei Xu, Yixuan Gan, Jing Zhou, Wenlu Li, Yi Wang, Zhong Chen, Di Wu

**Affiliations:** Zhejiang Collaborative Innovation Center for the Brain Diseases with Integrative Medicine, Zhejiang Key Laboratory of Neuropsychopharmacology, School of Pharmaceutical Sciences and School of Basic Medical Sciences, The Fifth Affiliated Hospital (Huzhou Central Hospital), Zhejiang Chinese Medical University, Hangzhou, China.

**Keywords:** ischemic stroke, biomimetic nanomaterials, polydopamine, inflammatory cascade, acidosis-responsive.

## Abstract

**Rationale:** Although reperfusion has been established as the main strategy for stroke treatment, it frequently causes irreversible secondary injury with dynamic pathological changes. The ischemic microenvironment in the brain continues to deteriorate after reperfusion, resulting in progressive expansion of the injured area and subsequent neuronal death. Therefore, it is urgent to find a therapeutic strategy for long-term management of ischemic stroke at post-reperfusion stages.

**Methods:** In this study, we designed an inflammation cascade-directed therapy using a biomimetic polydopamine nanosystem (MPP-B@MM). The synthesis of the nanosystem was confirmed by transmission electron microscopy, scanning electron microscopy, dynamic laser scattering, proton nuclear magnetic resonance, and so forth. The capability of blood-brain barrier crossing was investigated by cellular study with fluorescence imaging. Meanwhile, reactive oxygen species assay, apoptosis detection, and flow cytometry were employed to evaluate intracellular anti-apoptotic effects of the nanosystem. For *in vivo* evaluation, a middle cerebral artery occlusion model was established. Anti-stroke efficacy and mechanism of action of the nanosystem were assessed through multiple analytical methods, including behavioral tests, immunohistochemical staining, mRNA sequencing, and blood biochemical analysis.

**Results:** *In vitro* experiments demonstrated that MPP-B@MM exhibited superior cellular uptake and blood-brain barrier crossing, significantly attenuated mitochondrial damage, and rescued injured neurons. Comprehensive* in vivo* studies, spanning both acute and chronic phases, confirmed the superior long-term therapeutic performance of the nanosystem. Importantly, mRNA sequencing and pharmacological intervention experiments demonstrated that Homx1 served as the predominant molecular target for acidosis-responsive drug release.

**Conclusions:** This tailored nanosystem demonstrated acute neuroprotective effects during the initial phase of ischemic stroke. As inflammation progressed, the acidosis-responsive motif in the nanosystem could serve as an on-demand therapeutic method, spatiotemporally increasing neurotrophic factor expression at the stroke lesion, which significantly contributed to brain recovery.

## Introduction

Stroke is a lethal cerebrovascular disease that widely threatens public health. Ischemic stroke accounts for approximately 87% of all stroke incidents with high mortality and disability rates 1-4. Characterized by acute or persistent cerebral ischemia and hypoxia, stroke could lead to irreversible brain damage due to disrupted blood circulation and reduced supply of oxygen, glucose and ATP 5,6. Currently, recombinant tissue-plasminogen activator, approved by the US Food and Drug Administration, is the only thrombolytic strategy in emergency medical care 7. However, sudden restoration of nutrients and oxygen could trigger an inflammation cascade, leading to severe reperfusion-induced injury 8-12. The ischemic microenvironment continues to deteriorate under dynamic pathological changes, resulting in an expanded injured area and irreversible neuronal death 13, 14. So far, a number of therapeutic approaches, including neuroprotective agents, have been developed to facilitate the recovery of neurological dysfunction in long-term management of stroke 15-19. Nevertheless, blood-brain barrier (BBB) acts as a major obstacle, preventing nearly all therapeutic agents from entering the brain 20, 21. Although some studies reported that BBB leakage might allow passive drug accumulation in the brain lesion 22-24, the uneven distribution of such leakage undermines the accuracy of drug delivery. Thus, efficient and targeted drug delivery to the ischemic lesions remains a challenging task in the fields of pharmacy and biomedicine.

The inflammatory cascade is a spatiotemporally dynamic process that persists over the long-term post-ischemic period, involving a number of brain cells and inflammatory mediators 25-29. From a temporal perspective, the post-stroke inflammation cascade reaction can be roughly divided into acute (occurring within minutes), subacute (within hours), and chronic stages (days to months) 30-32. During the initial phase, the ischemic core exhibits an immediate surge in reactive oxygen species (ROS), including hydroxyl radical (·OH), superoxide anion radical (O_2_^·-^), hydrogen peroxide (H_2_O_2_), etc. 33-36. Several hours later, resident immune cells (e.g., macrophage) become activated, and peripheral leukocytes could infiltrate into the ischemic tissue, collectively releasing a number of pro-inflammatory factors 37-41. These biological processes accelerate the deterioration of stroke brain, leading to acidosis, calcium overload, and oxidative stress, which ultimately contribute to BBB leakage, brain edema, neuronal death, and etc 42-44. From a spatial perspective, post-stroke metabolic burden is unevenly distributed throughout the brain 45. For instance, tissue pH at the ischemic core becomes as low as 6.0 due to increased accumulation of lactate and pCO_2_, while in the ischemic penumbra, the pH value is slightly higher, ranging from 6.0 to 6.5 46-49. These acidified lesions, where a majority of neuronal death occurs, are crucial therapeutic targets for long-term recovery of brain functions in comparison with other brain regions.

Adaptive strategies have been reported in various diseases, including spinal cord injury, rheumatoid arthritis, and some stroke models. These systems dynamically target pathological signals (e.g., pH, ROS, or enzymatic activity) to achieve spatiotemporal control of drug delivery 50-52. However, current approaches face significant limitations, such as deficiencies in temporal and spatial control and a limited treatment time window. Previously, our team proposed an adaptive sequential treatment strategy based on the dynamic progression following neuroinflammation at the early stage. This strategy specifically targets the management of injuries resulting from ischemia-reperfusion. However, this approach specifically targets management of the early stage of ischemia-reperfusion injury, with no current investigations exploring its potential for functional neuronal recovery in the chronic post-stroke period 53. Furthermore, it is essential to explore methods for promoting neuronal repair and enhancing motor function at the later stage of recovery. Therefore, it is urgent to establish an adaptive strategy for long-term management of ischemic stroke with both spatially and temporally dynamic pathological changes.

It has been widely recognized that brain-derived neurotrophic factor (BDNF) is the most abundant neurotrophic factor in human body. Upon binding to its receptor Tropomyosin receptor kinase B (TrkB), BDNF activates the intracellular signaling domain and promotes autophosphorylation of TrkB, subsequently triggering the Ras-MAPK signaling pathway. This could lead to the phosphorylation of cAMP response element-binding protein (CREB) at its serine residue. Phosphorylated CREB enhances neuronal survival, synaptic plasticity, and neurogenesis by upregulating the expression of both BDNF gene and anti-apoptotic protein BCL-2 gene 54. Furthermore, BDNF improves neuron survival in brain, as well as facilitates growth, regeneration, and creation of newborn neurons and synapses, which is beneficial for stroke brain recovery 55. However, several limitations of BDNF, including its susceptibility to enzymatic degradation and difficulty in traversing the BBB restrict its potential for further application.

**Scheme 1:**
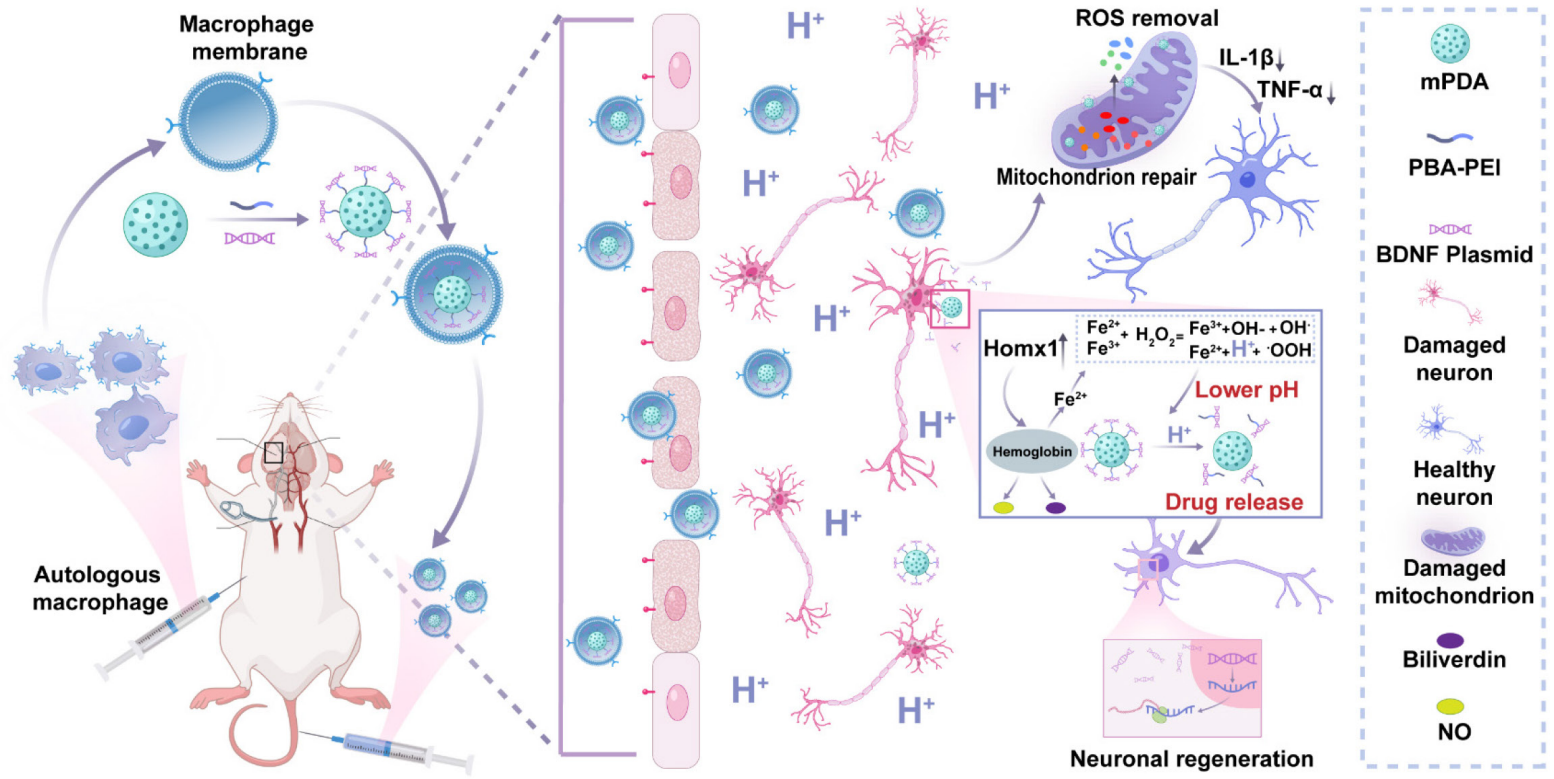
Inflammatory cascade-directed MPP-B@MM nanosystem for long-term management of ischemic stroke. Schematic diagram of preparation process and therapeutic performance of MPP-B@MM nanoparticles.

Hence, in this study, we proposed an inflammatory cascade-directed strategy for long-term management of ischemic stroke (Scheme 1). An acidosis-responsive plasmid delivery system with efficient ROS scavenging was established by preparing mesoporous polydopamine (mPDA) and loading cationic polymer/plasmid complexes. Mussel-inspired polydopamine has been demonstrated as an ideal ROS scavenger, and the tailored mesoporous nanostructure further improved radical removal performance by providing increased reactive sites 56-60. Notably, the complexes of interest could be conjugated onto the mesopores via catechol/boronate complexation chemistry and subsequently released in response to acidified conditions. To facilitate the targeted delivery to ischemic areas, we constructed a biomimetic targeting strategy via autologous macrophage membrane (MM) coating of drug-loaded mPDA nanosystem (abbreviated as MPP-B@MM). Following innate inflammatory cascade responses, specialized circulating cells including macrophages are recruited to the ischemic lesion from the periphery. The receptors (CD44 and CD11b) on macrophage membrane can interact with the overexpressed intercellular adhesion molecule-1 (ICAM-1) and P-selectin on lumen side of stressed vascular endothelial cells 61-63. Therefore, such a nanoplatform is expected to exert therapeutic effects at different post-stroke phases, providing an adaptive strategy in fight against the spatiotemporally dynamic stroke injuries.

## Materials and Methods

### Materials

Dopamine hydrochloride (DA·HCl) and Pluronic F127 were purchased from Sigma (Shanghai, China). 4-carboxyphenylboronic acid (PBA), 3, 3′, 5, 5′-Tetramethylbenzidine (TMB), and 1, 3, 5-Trimethylbenzene were obtained from Shanghai Aladdin Biochemical Technology Co., Ltd (Shanghai). Polyetherimide (PEI) (Mw = 1800) was purchased from Shanghai Yuanye Bio-Technology (Shanghai, China). Fluo-4 AM, Terminal deoxynucleotidyl transferase-mediated dUTP nick end labeling (TUNEL) apoptosis assay kit, enhanced mitochondrial membrane potential assay kit with JC-1, 1-(3-Dimethylaminopropyl)-3-ethylcarbodiimide (EDC), and 4',6-Diamidino-2-phenylindole (DAPI) were purchased from Beyotime (Shanghai, China). 1,1-diphenyl-2-picryl-hydrazyl (DPPH·) was bought from MCE (Beijing, China). Jet PRIME transfection reagent was purchased from Yeasen Biotech Co., Ltd (Shanghai, China). Indocyanine Green (ICG) was purchased from Shanghai Macklin Biochemical Co., Ltd (Shanghai, China). Mouse Peripheral Blood Lymphocyte Separation Kit was bought from Biosharp (Anhui, China). BDNF plasmid was bought from Baoke Biotech Co., Ltd (Shanghai, China). Picogreen dsDNA quantitation reagent was bought from Mkbio (Shanghai, China). Endo-free plasmid mini kit II was purchased from OMEGA (America). N-Hydroxy succinimide (NHS) was obtained from Sigma-Aldrich (America). 2, 3, 5-triphenyltetrazolium chloride (TTC) staining solution, ROS detection kit, DCFH-DA, DHE, thioglycollate broth, and Trypsin-EDTA Solution (0.25%) were obtained from Solarbio (Shanghai, China). DPPH·and Zinc protoporphyrin were purchased from Med Chem Express (America). Tumour necrosis factor-α (TNF-α), Interleukin-1β (IL-1β), and BDNF Enzyme-linked immunosorbent assay (Elisa) kits were purchased from Meibiao Biotechnology (Jiangsu, China). Optimal cutting temperature compound (OTC) was bought from Epredia (America). (3-ethylbenzothiazoline-6-sulfonic acid) (ABTS^+·^) was bought from Merck (Beijing, China). The Dulbecco's modified Eagle's medium (DMEM) and fetal bovine serum (FBS) were obtained from Gibco (Australia). Homx1 rabbit mAb, CD11b rabbit mAb, and CD44 rabbit pAb were bought from Proteintech (Wuhan, China). LC3 pAb was bought from Sigma (America). P62 rabbit mAb and Mouse IIgG (Total) Elisa Kit were brough Abclonal (Wuhan, China). PE anti-mouse/human CD44 Antibody, and APC anti-mouse CD62L Antibody were bought from Biolegend (America). Iba-1/AIF-1 (E4O4W) XP® Rabbit mAb, GFAP (GA5) Mouse mAb, and anti-NeuN were bought from Cell Signaling Technology (America).

### Animals

C57BL/6J male mice (8 weeks, 20-25 g) were purchased from Zhejiang Chinese Medical University Laboratory Animal Research Center. All animal treatments or procedures are carried out under the guidelines formulated by the Laboratory Animal Center of Zhejiang Chinese Medicine University (IACUC-20230717-08).

### Cell culture

Human neuroblastoma SH-SY5Y cell lines, murine microglial BV2 cell lines, and brain-derived Endothelial.3 cell lines (bEnd.3), and Jurkat, Clone E6-1 cell lines were purchased from ATCC (CRL-2299, Manassas, VA, USA), and incubated in DMEM medium with 10% FBS and 1% penicillin/streptomycin at 37 ºC and 5% CO_2_ incubator.

### Preparation and characterization of nanoparticles

#### Preparation of mPDA

According to previous literature reports, mPDA was prepared with a slight modification 53. Briefly, 15 mg DA·HCl aqueous solution and 50 mg Pluronic F127 ethanol solution were mixed for 1 min, and then 30 μL 1, 3, 5-trimethylbenzene was added. The mixture was ultrasonicated to form an oil-in-water emulsion. After adding 100 μL NH_3_^.^H_2_O, the mixture was stirred vigorously for 1.5 h, and the products were collected by centrifugation and washed with 50% ethanol three times.

#### Preparation of mPDA-PBA-PEI and mPDA-PEI

The responsive fragment of PBA-PEI was synthesized through an EDC/NHS reaction. Briefly, EDC (160 mg) and NHS-Sulfo (240 mg) were dissolved in MES buffer (0.1 M, pH = 5.2), followed by the addition of PBA (33.2 mg/mL in DMSO) to the mixture under magnetic stirring for 15 min. The pH was then adjusted to 8 using triethylamine. After that, 216 mg of PEI was added and stirred at room temperature for 24 h. The resulting product was dialyzed against aqueous solution for another 24 h (MWCO = 3500 Da).

mPDA and PBA-PEI were dissolved in Tris-HCl buffer (10 mM, pH = 8.5) at a mass ratio of 1:8. After being stirred overnight, the pH-responsive fragment of mPDA-PBA-PEI (abbreviated as MPP) was obtained through centrifugation and purification. Besides, PEI was used to synthesize mPDA-PEI (abbreviated as MP), serving as a non-responsive control.

#### Preparation of MPP-B

BDNF and MPP were combined in MES buffer solution (0.1 M, pH = 6.0) at varying nitrogen-phosphorus (N/P) ratios. The mixture was then shaken on a shaker set to 37 °C at 180 rpm for 30 min. Upon completion of the reaction, the resultant complexes were isolated via centrifugation at 8,000 rpm for 10 min. The supernatant was carefully removed, resulting in the acquisition of MPP-B. The non-responsive drug delivery system (MP-B) was prepared according to the same methodology but using MP instead of MPP.

#### Preparation of macrophage membrane

C57BL/6J mice were injected intraperitoneally with 3% thioglycollate broth once a day for three days to induce macrophage recruitment. Following the treatment period, mice were anesthetized, and the peritoneal cavity was irrigated twice with pre-cooled phosphate-buffered saline (PBS). Subsequently, intraperitoneal fluid was withdrawn and centrifuged at 300 rpm for 5 min. The isolated cells were obtained after discarding the supernatant and cultured overnight in the CO_2_ incubator. The adherent macrophages were collected and resuspended with hypotonic Tris-magnesium buffer to rupture the cells overnight at 4 ºC. The extracted cell membranes were obtained by centrifugation at 4,000 rpm for 20 min, and the pellet was resuspended in distilled water. The macrophage membrane was stored at -80 °C for further use after the protein content was quantified.

#### Preparation of MPP-B@MM

MPP-B@MM was prepared by mixing MPP-B and MM at a mass ratio of 2:1, followed by stirring at 4 ºC for 30 min. After that, the mixture underwent repeated extrusion 30 times using a filter membrane extruder with a pore size of 200 nm. Finally, the successful encapsulation of MM on MPP-B was examined by sodium dodecyl sulfate-polyacrylamide gel electrophoresis (SDS-PAGE). As a non-responsive control, MP-B@MM was obtained using the same procedure as MPP-B@MM except that MP-B was used instead of MPP-B.

### *In vitro* examination of BDNF release from MPP-B

MPP-B was dissolved in PBS buffer at various pH (6.0, 6.5, 7.0, 7.4), and shaken at 100 rpm/min at 37 ºC. At pre-determined times, the supernatant was collected through centrifugation. The concentration of released BDNF plasmid in the supernatant was quantified using the Picogreen dsDNA quantitation assay according to the manufacturer's protocol.

### Free radical scavenging capacity of MPP-B@MM

The free radical scavenging ability of MPP-B@MM was evaluated using DPPH, ABTS, and TMB assays. In the DPPH assay, a 0.5 mM DPPH solution was mixed with MPP-B@MM at concentrations of 25, 50, 75, and 100 μg/mL. Then the mixtures were incubated in darkness at room temperature for 30 min prior to spectrophotometric analysis at 517 nm.

In the ABTS assay, a working solution of ABTS^+·^ was prepared by mixing ABTS solution (7.4 mM) with an equal volume of 2.6 mM potassium persulfate solution. After that, the mixture was incubated in the dark at room temperature overnight. Subsequently, MPP-B@MM at concentrations of 25, 50, 75, and 100 μg/mL were reacted with the ABTS^+·^ working solution for 6 min, after which absorbance measurements were conducted at 734 nm.

In the TMB assay, a mixture containing Fe^2+^ (0.1 mM) and H_2_O_2_ (1 mM) was prepared, followed by sequential addition of MPP-B@MM solutions at concentrations of 25, 50, 75, and 100 μg/mL. The reaction proceeded for 15 min at room temperature before measuring absorbance at a wavelength of 652 nm.

### Cellular uptake

ICG was utilized to label MPP-B@MM or MPP-B for *in vitro* cellular uptake evaluation. MPP-B@MM or MPP-B (1.5 mg) were reacted with ICG (1.5 mg) for 24 h in dark. After centrifugation, ICG-labeled MPP-B or MPP-B@MM was obtained, washed, and dispersed in PBS for further use. SH-SY5Y cells were seeded in 24-well plates at a density of 5 × 10^4^ cells/well, and cultured overnight. MPP-B or MPP-B@MM (ICG concentration: 20 μg/mL) were co-cultured with the cells for 2, 4, 8, and 12 h, respectively. After that, the cells were washed with PBS three times, fixed with 4% paraformaldehyde for 30 min, followed by DAPI staining for 15 min, and observed by a virtual digital slice scanning system (Leica, Germany).

The bEnd.3 cells were inoculated in 6-well plates at an initial density of 5 × 10^5^, and cultured overnight. MPP-B@MM (ICG concentration: 20 μg/mL) was co-cultured with the cells for 2, 4, and 6 h. After that, the supernatant was discarded, cells were washed with PBS three times, and the cells were resuspended in PBS, followed by recording immediately using a flow cytometer (Beckman, Coulter), and then analyzed using CytExpert software based on 10,000 gated events.

### *In vitro* blood-brain barrier transcytosis study

The bEnd.3 cells were seeded in the upper chamber of a 24-well transwell plate at a density of 3 × 10^4^ cells/well, with a pore size of 0.4 μm in the upper chamber membrane. The trans-endothelial electrical resistance (TEER) value of bEnd.3 cells was measured every two day, until it reached and stabilized above 150 Ω cm^2^, confirming the successful construction of BBB model. A205848 (P-selectin inhibitor, 25 nM), PSI-697 (adhesion factor inhibitor, 100 µM), and Compound C (ZO-1 inhibitor, 50 µM) were pre-treated bEnd.3 cells for 2 h. Subsequently, MPP-B@MM (ICG concentration: 20 μg/mL) was added to the upper chamber. After incubation for 4 h, the upper chambers were taken out, and fluorescence intensities of the upper and lower chambers were observed with an *in vivo* imaging system (IVIS Lumina Series Ⅲ, America).

### Intracellular pH detection in SH-SY5Y cells

The pHrodo Green AM indicator dye effectively penetrates cell membranes and remains intact following non-specific esterase cleavage. Free fluorophores (AM) exhibit a weak fluorescence in the cytoplasm of neutrophils. During endocytosis, these fluorophores become encapsulated within endosomes that mature into acidic lysosomes. In this acidic environment, the molecular structure of the fluorophores undergoes changes that significantly enhance their fluorescence quantum yield, resulting in the generation of intense green fluorescence. These intracellular fluorescent probes serve as effective tools for detecting cellular pH changes. SH-SY5Y cells were initially seeded at a density of 5 × 10^5^ on confocal dishes. After 24 h incubation, the cells were divided into five groups: oxygen-glucose deprivation (OGD), oxygen-glucose deprivation/reoxygenation (OGD/R)-6 h, OGD/R-12 h, OGD/R-24 h, and normal condition as a control. Following various treatments, discarded the cell supernatants and washed the cells three times with PBS. Subsequently, a working solution (pHrodo™ Green AM: Power Load™: PBS = 1: 10: 1000) was added and incubated for 30 min. After incubation, wash the cells three times with PBS and observe them using Confocal Laser Scanning Microscopy (CLSM) (FV3000, Japan).

### Intracellular anti-apoptotic capacity, mitochondrial membrane potential, and intracellular calcium ion content detection

The SH-SY5Y cells were inoculated on a 24-well plate at a density of 5 × 10^4^ cells/well for 12 h. Following OGD treatment for 4 h, cells were incubated with different groups for 24 h (Normal, OGD, BDNF plasmid, MPP@MM, MPP-B@MM). After that, the cells were subjected to specific fluorescent probes to evaluate various cellular parameters: DCFH-DA, TUNEL, JC-1, or Fluo-4 for detection of intracellular ROS, apoptotic situation, mitochondrial membrane potential, and calcium ion, respectively.

### Immunotoxicity test

For cell proliferation studies, macrophage membrane and MPP-B@MM were co-incubated with Jurkat and CloneE6-1 cells (used for investigating acute T-cell leukemia and T-cell signaling) for 24, 48, and 96 h, respectively. Subsequently, newly proliferated cells were labeled with CFSE fluorescent probe (used for detecting cell proliferation and division), and analyzed via flow cytometry.

C57BL/6J mice were randomly assigned to experimental groups. Therapeutic agents (MM, MPP-B@MM) were administered via intravenous injection on days 1 and 3, respectively. Peripheral blood was collected from the orbit on day 21. Then, add 3 mL of mouse lymphocyte isolation solution and centrifuged (500 g, 20 ℃, 30 min) to isolate mononuclear cells from the middle layer. The isolated cells were diluted with 10 mL of PBS and centrifuged (250 g, 20 °C, 10 min). After discarding the supernatant, the cell pellet was resuspended in 200 μL of PBS. Then add 1 μL PE anti-mouse/human CD44 antibody, and APC anti-mouse CD62L antibody and incubate for 30 min before analysis by flow cytometry.

The blood samples of mice were collected using the above-mentioned method, followed by centrifugation at 1,000 g for 10 min to obtain serum. Total immunoglobulin G (IgG) levels in each group were measured utilizing the mouse IgG (Total) Elisa kit according to the manufacturer's protocol.

### Detection of the transfection ability of MPP@MM

Blank plasmids labeled with GFP fluorescence were utilized as model plasmids, and commercially available jetPRIME reagents were used as a positive control. SH-SY5Y cells were seeded at an initial density of 5 × 10^4^ in a 24-well plate. After 24 h of incubation, the cells were divided into OGD group and normal control group. Subsequently, DNA plasmids with MPP@MM or jetPRIME were mixed and added into each well with the appropriate ratio according to the protocol. After transfection, the cells were captured via CLSM to evaluate the transfection efficiency across different experimental conditions.

### Western blot analysis

The SH-SY5Y cells were inoculated in 6-well plate with a density of 5 × 10^5^ cells/well and cultured for 12 h. After OGD treatment for 4 h, cells were incubated with different groups for 24 h. Protein concentration was measured with a BCA protein detection kit. Samples containing equal amounts of proteins were then added to 10% SDS polyacrylamide gels for electrophoretic separation, followed by membrane transfer, and finally incubated for 2 h using 5% bovine serum albumin. At the end of the blocking period, the samples were incubated overnight at 4 ºC with primary antibodies: β-actin (1:5000), LC-3 (1:800), P62 (1:1000), and then incubated at room temperature with HRP-conjugated secondary antibody for 1 h. Finally, the protein bands were visualized by a gel imaging system, and band gray intensities were quantified and measured by Image J software.

### Middle cerebral artery occlusion/reperfusion model establishment

In this study, a middle cerebral artery occlusion/reperfusion (MCAO/R) model was established by a thread embolization method. C57BL/6J mice were anesthetized with pentobarbital and positioned supinely. An incision was then made along the midline of the neck. The inner edge of the sternocleidomastoid muscle was identified and separated from the underlying fascia. Subsequently, ligation was performed at the proximal end of the heart, 4 mm distal to the bifurcation of the common carotid artery (CCA). A small incision was made to insert a thread plug into the middle cerebral artery (MCA). 1 h after placement in the MCA, the plug was removed to initiate reperfusion. Immediately afterward, mice were intravenously injected with Saline, BDNF, MPP@MM (without BDNF plasmid), and MPP-B@MM (BDNF: 40 μg).

### TTC staining

After 24 h, the homogeneously cut brain tissues were put into 2.5% TTC solution and incubated at 37 ºC in the dark for 15 min. Then 4% paraformaldehyde was added for fixation for 30 min. At the end of the staining procedure, the brain tissue sections were photographed with a scanner for further analysis.

### Cerebral edema assessment

In this study, a wet/dry method was used to detect the degree of brain tissue edema. Briefly, the brain tissues of mice after 24 h of MCAO/R were collected and divided into infarcted hemisphere, contralateral hemisphere, cerebellum, and then the weight of these three parts was measured and recorded as the wet weight. Subsequently, the tissues were placed in an oven at 75 °C and dried for 48 h, and then the weight of these three parts was measured and recorded as the dry weight. Finally, the brain water content was calculated using the formula: Brain water content = (Wet weight - Dry weight)/Wet weight × 100%.

### Detection of inflammation

IL-1β and TNF-α are two typical pro-inflammatory factors. Therefore, in this study, we measured the inflammation level of infarcted hemisphere tissues by Elisa. After experimental testing, fresh mouse tissues were collected, and the supernatants were taken by homogenization and centrifugation (3,000 rpm, 10 min) for the detection of inflammation factors according to the Elisa kit instruction.

### ROS and TUNEL staining in brain

The treated mice were sacrificed after reperfusion for 24 h. *In vivo* ROS detection of brain slices was performed using the dihydroethidium (DHE) assay kit. Brain slices (15 μm thickness) were stained with DHE (20 μM) for 45 min at 37 ºC. In addition, 50 mg of fresh tissue was excised from infarcted hemisphere. Following homogenization of the tissue, the supernatant was subjected to centrifugation (100 g, 4 °C, for 3 min). Subsequently, 200 μL of the homogenized supernatant and 2 μL of DHE probe were added to a 96-well plate. The mixture was incubated at 37 °C in darkness for 30 min. The fluorescence intensity was measured (Ex/Em = 488/610 nm).

TUNEL staining was used to determine neuronal apoptosis in the brain. Brain slices (10 μm thickness) were collected and washed three times with PBS, and treated with 0.3% Triton X-100 for membrane breaking treatment. The slices were then incubated with TUNEL assay solution at 37 ºC for 60 min. Finally, the brain slices were covered with DAPI for nuclei staining and then photographed by a virtual digital scanning system.

### Determination of brain-derived nerve growth factor in the brain

After the experimental test, fresh infarcted hemisphere tissues of mice were collected, and the supernatant was taken by homogenization and centrifugation (3,000 rpm, 10 min) and assayed by Elisa kit.

### mRNA-Sequencing experiment

Tissues (three mice per group) were processed in a sterile RNase-free environment using calcium-free and magnesium-free 1 × PBS on ice. The total RNA from the brain tissues of mice from different groups (Sham, Saline) was extracted using TRIzol (Invitrogen). After enrichment and purification of the obtained mRNA, the samples were assessed by Personal Biotechnology Co., Ltd. (China). mRNA-Seq data was analyzed by Personal Biotechnology Co., Ltd. (China) and followed the company instruction.

### Behavioral test

In this study, neurological damage in different groups after MCAO/R was assessed by performing a series of behavioral tests. Mice were randomly divided into different groups (Sham, Saline, BDNF, MPP@MM, MPP-B@MM) (BDNF: 40 μg). On the day of the test, mice were acclimatized to the test chamber for 30 min (domestication phase). Stickers of uniform size and adhesion (0.3 × 0.4 cm^2^) were pasted on the medial side of the upper limb of the mice. Two parameters were recorded for each mouse: the time to first touch with the sticker and the time to completely remove the sticker.

In addition, we detected the recovery of locomotor ability in mice by the rotarod test. Mice were randomly divided into different groups (Sham, Saline, BDNF, MPP@MM, MPP-B@MM) (BDNF: 40 μg), which had been acclimatized to the environment three days before the test. On the day of the test, the mice were acclimatized in the test chamber for at least 15 min (acclimatization phase) and held in equilibrium on the rotameter for at least one minute. During the test, the cylinder was rotated from 0 to 40 rpm for 2 min, then at a fixed speed for another 3 min. Each mouse was tested eight times (20 min every time), with the latency to fall and maximum speed reached on the rod were recorded during each trial.

In the open-field test, a box with a white background was used to test each mouse for 10 min. Average speed, total distance of their total motion, and speed and distance of their movement in the central region were recorded to evaluate their spontaneous activities.

Immunofluorescence staining: Brain tissues were first fixed with 4% paraformaldehyde and dehydrated with 30% sucrose solution at 4 ºC overnight. The brain slides (30 μm thickness) were washed three times with PBS, permeabilized with 0.1% Triton X-100 for 10 min, and blocked with 5% bovine serum albumin for 30 min. The brain sections were then incubated with primary antibodies at 4 °C overnight, including: rabbit anti-NeuN (1:500), rabbit anti-GFAP (1:500), rabbit anti-Iba-1 (1:500). After washed three times with PBS, the sections were incubated with Alexa Fluor 488/647 fluorescent conjugated secondary antibody (1:500) at room temperature for 2 h. Finally, the brain slices were covered with DAPI for nuclei staining and then photographed by a virtual digital scanning system.

### Statistical Analysis

All values are expressed as the mean ± SEM, and Prism software (version 10.1) was applied to perform the statistical analysis. One-way ANOVA analysis. Significance was determined at *p* < 0.05 (*p < 0.05, **p < 0.01, ***p < 0.001, ****p < 0.0001).

### Graphical Illustrations

Graphical illustrations were created with Biorender (biorender.com).

## Results

### Synthesis and characterization of MPP-B@MM nanoparticles

**Figure 1:**
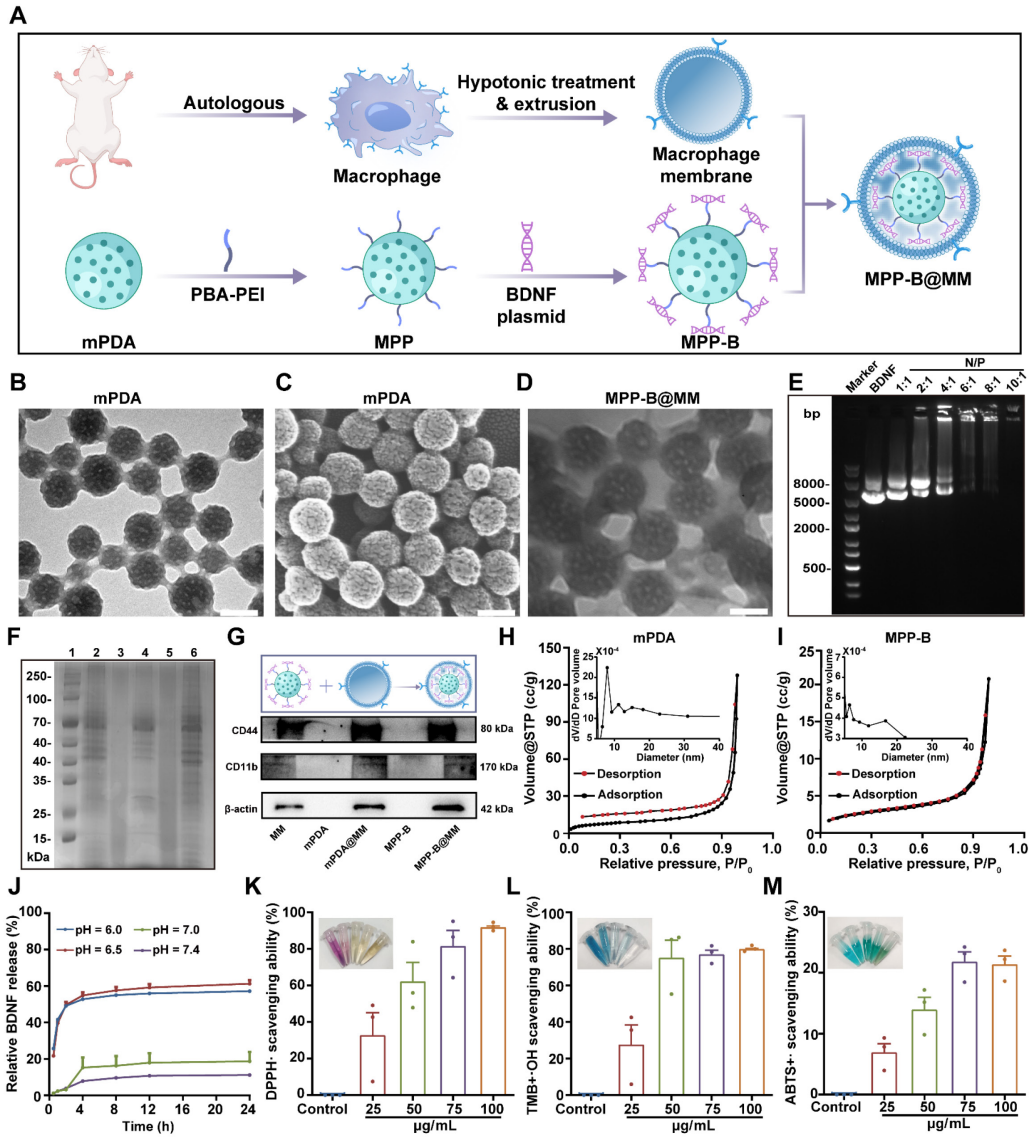
** Synthesis and characterization of MPP-B@MM nanoparticles. (A)** The synthesis process of MPP-B@MM. **(B)** The representative TEM image and **(C)** SEM image of mPDA. **(D)** The representative TEM image of MPP-B@MM. **(E)** Electrophoresis of BDNF loaded nanoparticle at various N/P ratios. **(F)** SDS-PAGE protein analysis of marker (1), MM (2), mPDA (3), mPDA@MM (4), MPP-B (5), and MPP-B@MM (6). **(G)** Western blot analysis of key proteins on MM, mPDA, mPDA@MM, MPP-B, and MPP-B@MM. N_2_ adsorption/desorption isotherms of **(H)** mPDA and **(I)** MPP-B. Inset: Pore size distribution of mPDA and MPP-B. **(J)** Cumulative drug release profiles in the presence of different pH (*n* = 3). **(K)** DPPH·, **(L)** ·OH, and **(M)** ABTS^+·^ radical scavenging effects of MPP-B@MM at different concentrations (*n* = 3). The data are presented as means ± SEM.

The biomimetic polydopamine nanosystem for inflammation cascade-directed therapy could be obtained by tandem synthesis of mPDA nanoparticles and drug loading, followed by macrophage membrane coating (Figure 1A). At first, an ethanol-F127-dopamine-water quaternary microemulsion was utilized for mPDA preparation. After adding ammonium hydroxide, mussel-inspired polydopamine monomers could undergo spontaneous self-polymerization within 1.5 h. As presented by transmission electron microscopy (TEM) and scanning electron microscopy (SEM), the particles exhibited a uniform distribution of particle size at a diameter of *ca.* 120 nm, as well as distinct mesopores throughout the particles (Figure 1B-C). The elemental mapping results in Figure S1 showed that a uniform distribution of C, O, N elements in mPDA from catechol and amine groups in polydopamine. Such mesostructured nanoparticles not only provide abundant readily provide more reactive sites but also simultaneously improve drug loading capacity.

To demonstrate the responsive drug release for neuronal regeneration and brain recovery, BDNF was selected as a proof-of-concept drug model as it encourages the growth and differentiation of newborn neurons in damaged brain. However, direct administration of BDNF is extremely low efficacy due to its quick degradation and poor brain accumulation during blood circulation. To address these challenges, plasmids of BDNF were used and could then be complexed with PEI (1.8 kDa) via electrostatic adsorption for improved transfection. To incorporate the acidosis-responsive motif for on-demand BDNF expression in the ischemic region, acid-sensitive bonds of catechol-phenylboronate were introduced onto mPDA surface. As shown in Figure S2A-B, the chemical structure of PBA-PEI was determined by ^1^H NMR. The characteristic peaks at 7 to 8 ppm and at 13 ppm were assigned to the aromatic rings and -COOH groups in PBA-COOH, respectively. Notably, the disappearance of the -COOH proton peak in the ¹H NMR spectrum of PBA-PEI confirmed the successful conjugation of PBA to PEI. Cationic PEI was modified with PBA and linked with mPDA via catechol-phenylboronate interactions. After BDNF plasmid complexation and cargo loading, the surface charge of drug-loaded nanoparticles (mPDA-PBA-PEI, abbreviated as MPP) showed a reversed surface charge from -22.5 ± 0.3 mV to 15.3 ± 0.3 mV (Figure S3). Ideal N/P ratio of PEI-BDNF complexation was optimized to 10:1 (Figure 1E).

Afterwards, biomimetic nanoparticles were obtained by cellular membrane wrapping. Successful membrane modification was confirmed by zeta potential analysis, which showed a reversal of the surface charge back to a negative state (-17.2 ± 0.2 mV), consistent with the negatively charged nature of cell membranes. Additionally, the results of SDS-PAGE analysis suggested that the membrane-coated and plasmid-loaded mPDA nanoparticles (MPP-B@MM) inherited functional proteins and oligonucleotides from original macrophages (Figure 1F). For example, overexpressed macrophagic CD44 and CD11b, which can recognize and bind with ICAM-1 and P-selectin on endothelial cells, were observed in MPP-B@MM. This feature is conducive to biomimetic targeting of the ischemic brain, laying a foundation for site-specific drug delivery (Figure 1G). Furthermore, to determine the optimal mass ratio of membrane to MPP-B, TEM was utilized to examine the integrity of the cell membrane coating as well as the dispersion of nanoparticles. The results indicated that at the mass ratio of 2:1, MPP-B@MM exhibited a uniform coating state with satisfactory dispersion. In contrast, employing either the ratio of 1:1 (excessive MM) or 3:1 (excessive MMP-B), caused significant nanoparticle agglomeration and incomplete membrane coating (Figure 1D and S5). Besides, Brunauer-Emmett-Teller (BET) analysis revealed that the surface area and the average pore diameter of mPDA before cargo loading were 27.33 m^2^/g and 28.9 nm, respectively. The BET surface area of mPDA is nearly 130-fold higher than non-mesoporous polydopamine (0.21 m^2^/g) (Figure S4). Comparatively, the drug-loaded MPP-B nanoparticles showed a notable reduced specific surface area and pore size, implying the anchored BDNF complexes inside the mesopores, occupying the porous space (Figure 1H-I). MPP-B nanoparticles demonstrated pH-responsive release of BDNF plasmids, as shown in Figure 1J. For instance, 57.2 ± 1.9% of loaded plasmids could be released in response to pH 6.0, which is at a similar pH level in the ischemic core. Importantly, interfering factors in the ischemic brain, such as hydrogen peroxide (H_2_O_2_) and matrix metallopeptidase (e.g., matrix metallopeptidase-2) have hardly any impact on drug encapsulation, further indicating the specificity and stability of as-designed acidosis-responsive drug release (Figure S6). ROS scavenging efficiency represents another crucial factor for ischemic stroke treatment. Three typical radicals, including DPPH, OH·, and ABTS^+·^ were selected to evaluate the removal performance of MPP-B@MM by scavenging assay kits. All three radicals could be readily quenched with MPP-B@MM as low as 25 μg/mL, owing to the intrinsic ROS scavenging capability of polydopamine and its tailored mesostructured (Figure 1K-M, Figure S7A-C).

### Neuroprotection of nanosystem in OGD/R models* in vitro*

**Figure 2:**
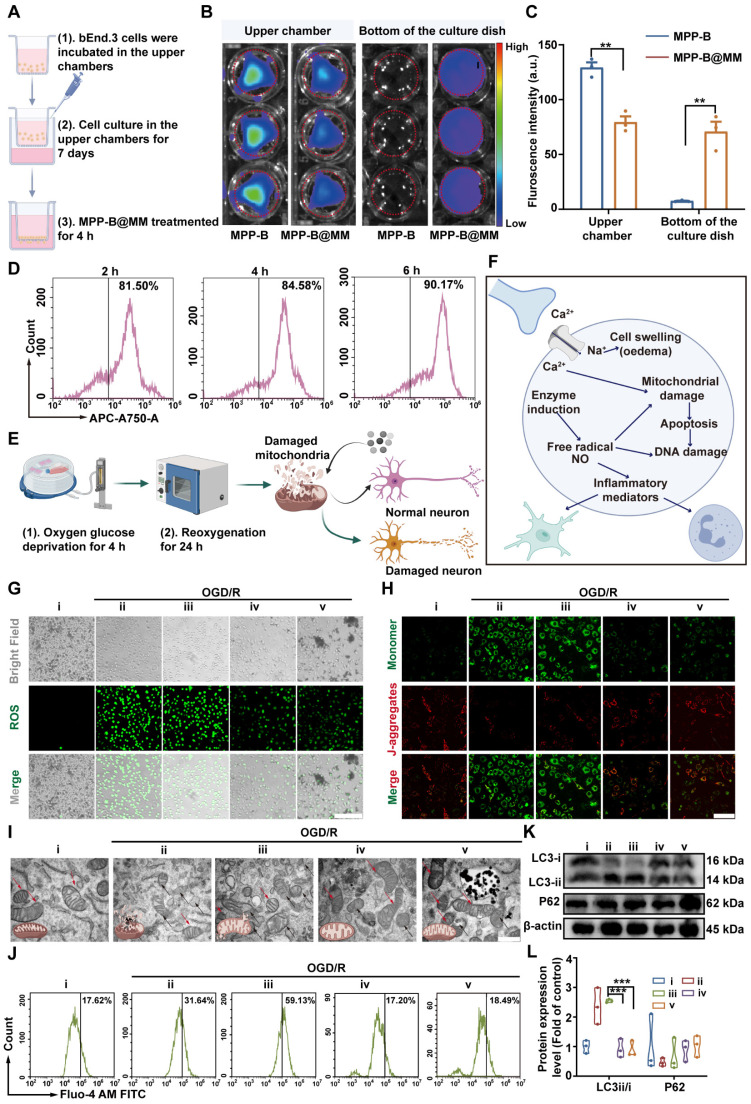
** Neuroprotection of nanosystem in OGD/R models *in vitro*. (A)** Scheme of transwell experimental flow (created with BioRender.com). **(B)** Fluorescence images of the lower and upper chamber after incubation of bEnd.3 with fluorescence-labeled MPP-B, MPP-B@MM for 4 h. **(C)**. Average relative fluorescence was estimated from images in B) (*n* = 3). **(D)** The uptake capacity of MPP-B@MM by bEnd.3 cells at 2, 4, and 6 h. **(E)** Scheme of constructing OGD/R model (created with BioRender.com). **(F)** Scheme of intracellular microenvironment changes after OGD/R (created with BioRender.com). **(G)** Representative images of intracellular ROS levels measured using DCFH-DA in SH-SY5Y cells. Scale bar, 200 μm. **(H)** Representative images of JC-1 fluorescence staining in SH-SY5Y cells. Scale bar, 200 μm. **(I)** The representative TEM image of the structure of mitochondria in SH-SY5Y cells. Scale bar, 100 μm. Red arrow: normal mitochondria; Black arrow: damaged mitochondria. **(J)** Comparison of intracellular calcium concentration by Fluo-4 AM dye. **(K)** Western blot analysis and **(L)** quantification of LC3 and P62 in SH-SY5Y cells (*n* = 3). i: Normal, ii: OGD/R + PBS, iii: OGD/R + BDNF, iv: OGD/R + MPP@MM, v: OGD/R + MPP-B@MM. The data are presented as means ± SEM. **p < 0.01, ***p < 0.001 via one-way ANOVA with Dunnett's multiple comparisons test.

The cytotoxicity of mPDA, MPP-B, and MPP-B@MM was assessed using the CCK-8 assay. The results indicated that all of them exhibited favorable biosafety, even at concentrations as high as 400 µg/mL (Figure S8). To evaluate the therapeutic effects of MPP-B@MM *in vitro*, a cellular study by using endothelial bEnd.3 and neuroblastoma SH-SY5Y cell lines was conducted. Firstly, transwell study was performed by cell seeding of bEnd.3 cells on the upper chamber to investigate BBB penetration. Until trans-epithelial electrical resistance reached above 150 Ω·cm^2^, the endothelial cells were treated with MPP-B@MM for 4 h (Figure 2A). Fluorescence imaging of upper and bottom chambers as shown in Figure 2B-C, indicated that the biomimetic membrane coating could significantly improve the penetration efficiency of nanoparticles (841%) through integrated endothelial monolayer. In contrast, nearly all unmodified nanoparticles remained in the upper chamber, confirming the critical role of MM coating in BBB penetration. Flow cytometry analysis of cellular uptake suggested 81.5% of bEnd.3 cells internalized ICG-tagged MPP-B@MM within 2 h. Extended incubation periods resulted in enhanced uptake efficiency (Figure 2D, Figure S9). To uncover the penetration mechanism, a monolayer of bEnd.3 cells was pre-treated with P-selectin inhibitor (A205848) and/or adhesion factor inhibitor (PSI-697) (Figure S10A-B). Compared with the control group, either kind of inhibitors alone suppressed the transportation from the upper to the bottom, and the combined treatment further intensified the inhibition (inhibition ratio, A205848: 61.5%, PSI-697: 58.3%, Combined: 85.1%). In addition, treatment with Compound C, an inhibitor of adenosine-monophosphate activated protein kinase (AMPK) that participates in tight junction regulation was used to treat the integrated endothelial monolayer. After the treatment, a higher ratio of biomimetic nanoparticles (66.3%) was observed at the bottom chamber, which in all indicated the MM coating strategy could largely facilitate the BBB penetration via multiple routes (Figure S10C-D).

To simulate brain ischemia and reperfusion-induced injury* in vitro*, an OGD/R model was conducted for the following study as shown in Figure 2E. At the initial stage of ischemic stroke, a burst of free radicals was released, causing mitochondrial damage, as well as excessive accumulation of lactic acid and hydrolysis of ATP to produce H^+^. These changes would lead to tissue acidification, which further triggered inflammatory responses (Figure 2F). Under such circumstances, MPP-B@MM showed enhanced therapeutic efficacy against ischemia-induced damage of neurons via efficient cellular uptake, strong ROS scavenging, and acidosis-responsive drug release. The macrophage-camouflaged strategy enabled higher uptake by SH-SY5Y cells, probably due to the elevated membrane fusion (Figure S11). Besides, effective plasmid transfection is the key to *in vivo* therapeutic function of BDNF treatment. Therefore, we used the non-viral vector MPP@MM complex to evaluate the transfection ability toward SH-SY5Y cells and used GFP plasmid instead of BDNF plasmid to observe its fluorescence intensity within the cells (Figure S12). This observation might be associated with the decrease in intracellular pH following OGD treatment. Hence, pHrodo dye was utilized to detect intracellular pH changes, the fluorescence intensity of the dissociative fluorescent group (AM) increases with the decrease of pH. As illustrated in Figure S13A-B, compared to normal cells, the intracellular pH exhibited a significant decline at OGD/R-0 h (without reperfusion), after which the pH gradually increased with prolonged reperfusion time. This indicated that immediate treatment after OGD could enhance the release of GFP plasmids and their intracellular expression.

### MPP-B@MM reduced OGD/R-induced apoptosis in SH-SY5Y cells

Importantly, mPDA nanoparticles demonstrated significant capability of intracellular ROS removal, irrespective of membrane coating (Figure 2G and S14). These positive effects could alleviate the OGD/R-induced mitochondrial damage, which was proved by the mitochondrial membrane potential assay kit with JC-1 staining (Figure 2H). JC-1 fluorescent dye could serve as a sensitive marker for mitochondrial membrane potential as it yields green fluorescence at 525 nm as monomers and shows a bathochromic-shift to the band at 590 nm as aggregates.

A higher red/green ratio was observed at the group of MPP-B@MM, which is at the similar level to untreated healthy cells, suggesting the protective effects of MPP-B@MM for neuron mitochondrial. Interestingly, the incorporation of acidosis-responsive modulus also contributed to mitochondrial protection due to the consumption of excessive acid (Figure S15). After 4 h of OGD, swollen and dissolved mitochondria as well as a loss of internal cristae, were observed in neuron cells as shown in TEM images (Figure 2I). In contrast, with the treatment of MPP-B@MM at 25 μg/mL, a majority of damaged mitochondria were rescued and their morphology was recovered to normal.

As is known, mitochondria is one of the major organelles for calcium storage and intracellular calcium concentration is a critical indicator of cellular oxidative stress. Thus, the OGD/R treatment will not only cause mitochondrial dysfunction, but also lead to calcium overload, as confirmed by Fluo-4 AM staining, which showed intracellular Ca^2+^ content increased from 17.6 ± 0.9% in the normal group to 31.6 ± 0.9% in the group of OGD/R with PBS treatment (Figure 2J and S16). The results showed that direct treatment with BDNF plasmids even worsens the situation. Fortunately, the treatments with MPP@MM and MPP-B@MM decreased the calcium overload to 17.2 ± 2.8% and 18.4 ± 3.5%, respectively. Meanwhile, autophagic activity under OGD/R treatment was evaluated by western blot assay of P62, a classic receptor of autophagy (Figure 2K-L). The reduced level of P62 at ischemic condition could be reversed by MPP-B@MM treatment, also accompanied by the decreased ratio of LC3-ii/LC3-i, implying the suppression of MPP-B@MM on autophagic activities. As a series of biological factors and processes, including cellular uptake, ROS level, mitochondria function and autophagic activity in neuron cells were improved, we tested whether OGD/R-induced cellular apoptosis was alleviated. It was indicated by TUNEL staining in Figure S17 that 59.5 ± 6.9% of TUNEL positive cells could be found after the treatment of OGD/R. But the cellular apoptosis was significantly reduced following incubation with MPP-B@MM, where the TUNEL positive cells decreased to just 6.1 ± 1.9%. The inflammatory response at the site of brain injury, exacerbated by hypoxia, is a significant characteristic of ischemic stroke. Our findings indicated that MPP-B and MPP-B@MM could effectively inhibit the expression of key pro-inflammatory cytokines, such as TNF-α and IL-1β. This suggests that MPP-B@MM exhibited a more potent inhibitory effect on OGD/R-induced neuronal death (Figure S18). All these results convincingly confirmed the BBB crossing ability and *in vitro* protective effects of MPP-B@MM, making it a promising candidate for therapeutic agents in the fight against ischemic stroke via inflammation cascade-directed therapy.

### *In vivo* brain protection of the nanosystem in middle cerebral artery occlusion/reperfusion mice model

**Figure 3:**
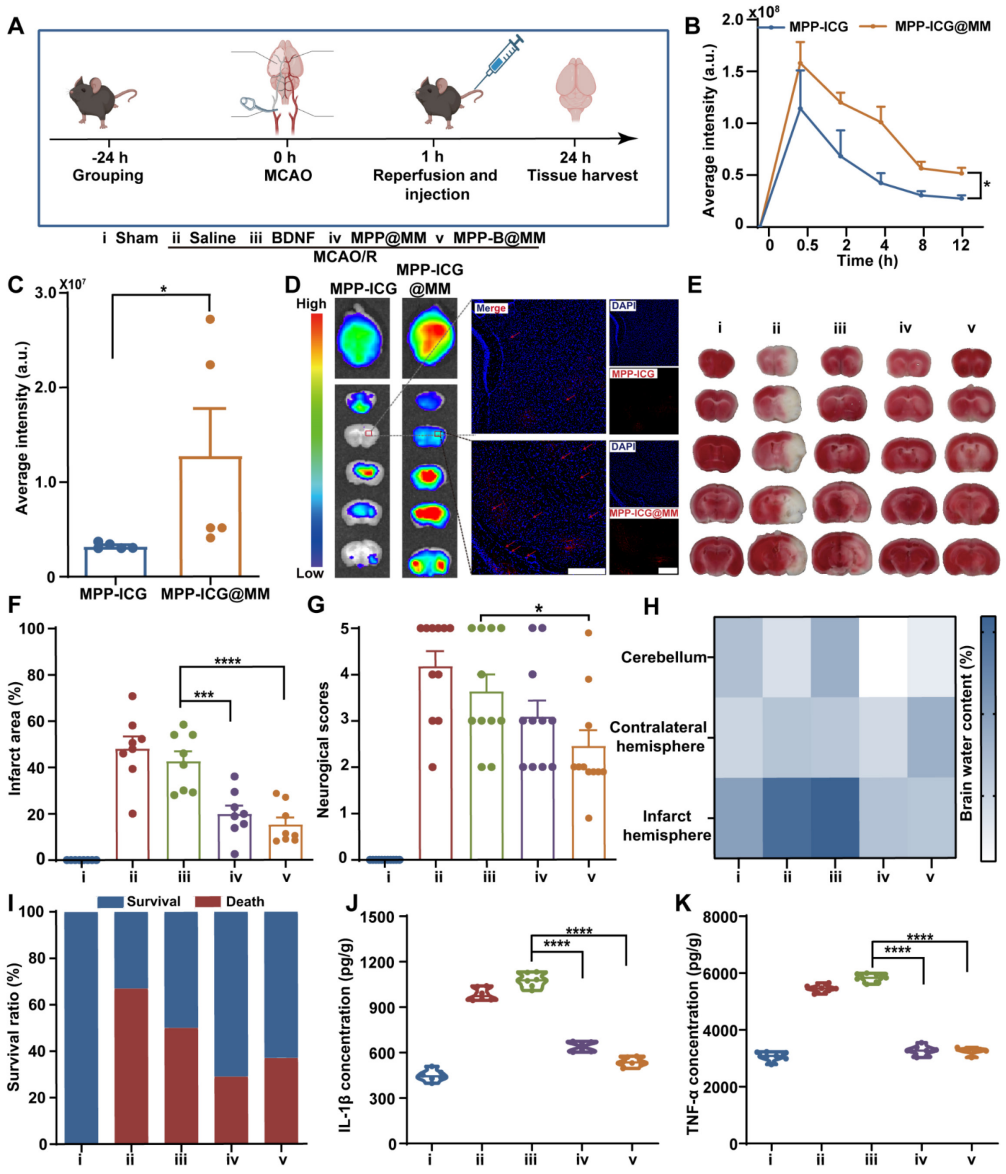
***In vivo* brain protection of the nanosystem in MCAO/R mice model. (A)** Schematic illustration of MCAO/R mice model establishment (created with BioRender.com). **(B)** The quantitative analysis of the accumulation of MPP-ICG and MPP-ICG@MM in the whole brain at different times by the *in vivo* imaging system (*n* = 6). **(C)** The quantitative analysis of the accumulation of MPP-ICG and MPP-ICG@MM in the whole brain at 0.5 h (*n* = 5). **(D)** Typical *ex vivo* fluorescence images of brain sections and slices from MPP-ICG and MPP-ICG@MM-treated mice. Scale bar, 200 μm. **(E)** Representative TTC staining images of brain slices from the mice with different treatments. **(F)** The quantified results of TTC staining (*n* = 8). **(G)** Neuronal function evaluation of the mice by neurological scoring (*n* = 11). **(H)** Brain water content in the infarcted hemisphere, contralateral hemisphere, and cerebellum was measured and statistically analyzed (*n* = 6). **(I)** Survival rate of the MCAO/R mice after different treatments in 24 h (*n* = 11). Serum inflammatory factors **(J)** IL-β and **(K)** TNF-α were detected by Elisa kit (*n* = 6). The data are presented as means ± SEM. *p < 0.05, ***p < 0.001, ****p < 0.0001 via one-way ANOVA with Dunnett's multiple comparisons test.

Then, to verify the therapeutic performance of biomimetic MPP-B@MM nanoparticles* in vivo*, a MCAO/R model was established according to our previous work 52. Briefly, the MCA was inserted with a filament to block the blood flow, and the filament was removed after 60 min to achieve ischemia reperfusion (Figure 3A). At the same time, different formulations, including Saline, BDNF, MPP@MM, and MPP-B@MM, were intravenously injected into each group. *In vivo* animal fluorescence imaging demonstrated that bionic MPP-ICG@MM nanoparticles exhibited more accumulation in the brain within 30 min than MPP-ICG (without macrophage membrane coating) (Figure 3B)*.* While the nanoparticles in blood circulation were excluded from the body via the liver and kidney at 12 h post-injection, those that had entered the brain remained at the lesions for a longer period (Figure S19-20). Compared with non-camouflaged nanoparticles, the membrane-coated MPP-ICG@MM nanoparticles showed nearly 400% higher brain accumulation, which confirmed the brain targeting ability (Figure 3C-D). Magnified fluorescence images showed penetrated nanoparticles in the ischemic penumbra.

### MPP-B@MM relieved neuronal apoptosis and loss in middle cerebral artery occlusion/reperfusion mice model

After reaching the site, the nanoparticles were expected to remove excessive ROS at the acute phase. Infarct volume results measured from TTC-stained brain slices indicated distinct injury protection by biomimetic nanoparticles (Figure 3E-F). In comparison with BDNF treatment (42.6 ± 4.4%), the membrane-coated MPP-B@MM nanoparticles showed higher therapeutic efficacy as the infarct volume decreased from 48.1 ± 5.2% in the Saline group to 15.3 ± 3.0% in the MPP-B@MM group. Behavior analysis also confirmed that the protective effects of acute injury are indicated by the reduced neurological scores from 4.2 ± 0.3 to 2.5 ± 0.3 (Figure 3G). In the meantime, administration of BDNF plasmids or MPP@MM could only alleviate neurological dysfunction to 3.6 ± 0.4 and 3.1 ± 0.3, respectively, indicating the significant difference. Further, brain water content in different brain regions was measured to evaluate the brain edema of MCAO/R mice after various treatments. Brain sections of cerebellum and contralateral hemisphere showed no significant difference between the groups of MCAO/R mice with Saline and other treatments (Figure 3H). In contrast, increased water content was observed in the infarct hemisphere of MCAO/R mice (73.3 ± 3.2%) compared with the Sham group (63.1 ± 1.4%). It was noted that no significant difference was observed between the groups of MPP-B@MM and MPP@MM (*p* = 0.4334, ns), indicating limited effects of BDNF plasmids on brain water content. It is suggested that the BDNF requires a longer period for higher expression. Administration of MPP@MM and MPP-B@MM could distinctively alleviate the ischemia injury due to ROS scavenging and brain protection properties of polydopamine nanoparticles at the acute phase (Figure S21). Thus, such protective effects of our biomimetic nanoparticles could rescue the MCAO/R mice and enhance the survival rate (Figure 3I). Further Elisa analysis of inflammatory factors in the brain sections proved a lowered pro-inflammatory microenvironment. Levels of both IL-1β and TNF-α were decreased after injection of MPP-B@MM, showing higher inflammation regulation capability than BDNF treatment alone (Figure 3J-K). Further analysis of cell loss at the region of hippocampus was conducted as shown in Figure S22. The ischemia injury caused severe cellular apoptosis, leading to obvious cell loss, whereas the group MPP-B@MM showed successful cellular rescue at the lesion. This effect of neuron protection was also confirmed by Nissl staining (Figure S23). After the treatment of MPP-B@MM, the number of Nissl positive cells was significantly increased and the cell distribution clearly returned to normal.

### Evaluation of brain protection and recovery of nanosystems in long-term management

**Figure 4:**
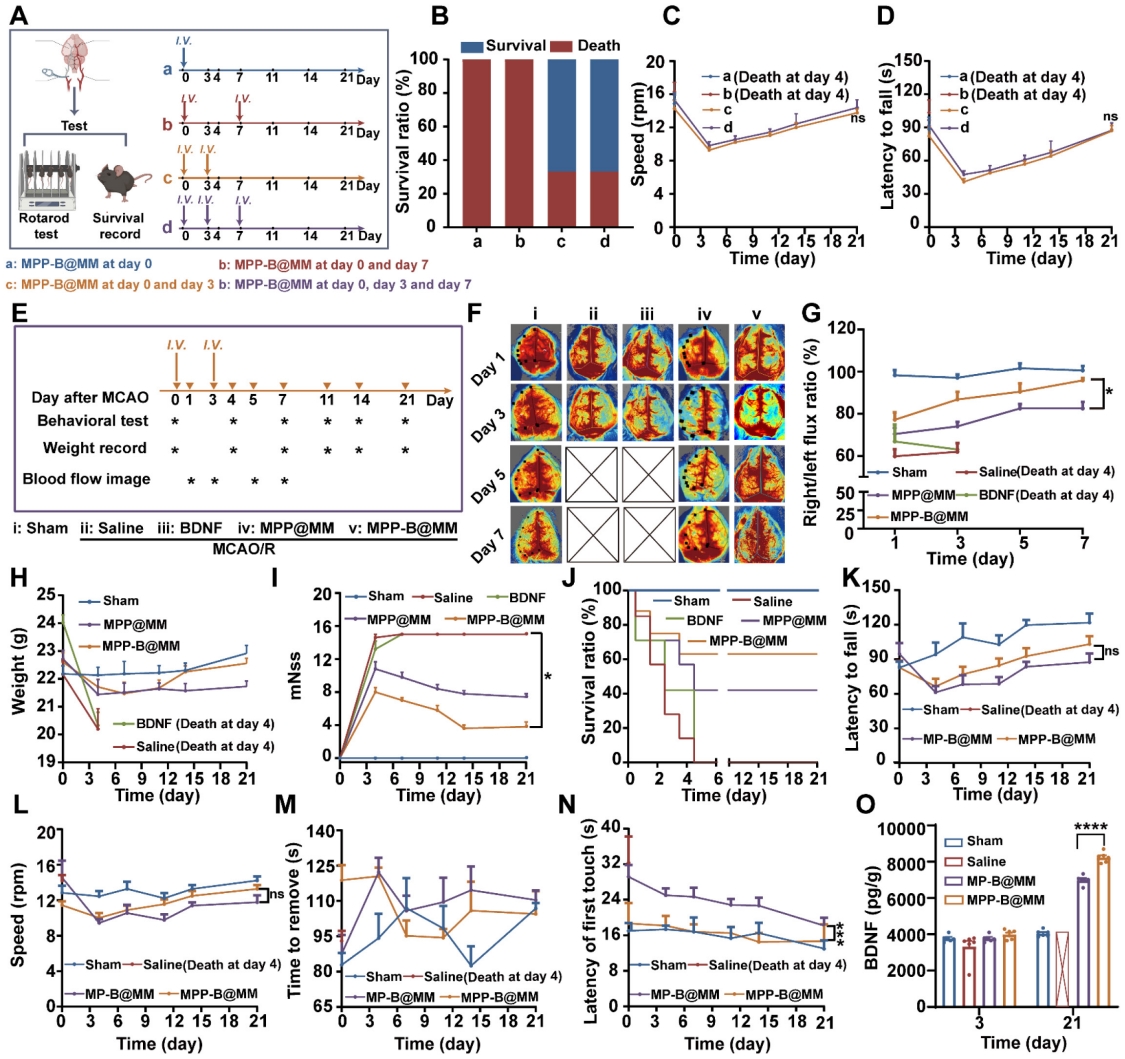
** Long-term neurorepair protection of nanosystems in MCAO/R mice model. (A)** Scheme of comparative study on the treatment in the group of a (MPP-B@MM at day 0), b (MPP-B@MM at day 0 and day 7), c (MPP-B@MM at day 0 and day 3), and d (MPP-B@MM at day 0, day 3 and day 7). **(B)** Survival rate of the MCAO/R mice with different treatments after 21 days (*n* = 6). **(C)** Rotarod fall speed and latency **(D)** in different groups were recorded (*n* = 6). **(E)** Time schedule of drug treatment and therapeutic evaluation. Inverted triangle: experimental day; Asterisk: test. **(F)** Typical images and **(G)** quantitative analysis results of cerebral blood test within 7 days after treatment in different groups (*n* = 4). **(H)** Weight records (*n* = 7) and **(I)** mNSS scores of different groups after treatment were obtained within 21 days (*n* = 5). **(J)** Survival rate of the MCAO/R mice after different treatments within 21 days. **(K)** Rotarod fall latency and **(L)** speed were recorded (*n* = 6). **(M)** The sticker removal time and **(N)** the latency of first touch were recorded (*n* = 6). **(O)** BDNF levels in the brain of different groups treatment on days 3 and 21 (*n* = 6). The data are presented as means ± SEM. ns: *p* > 0.05, *p < 0.05, ***p < 0.001 via one-way ANOVA with Dunnett's multiple comparisons test.

While the abovementioned results proved satisfactory therapeutic efficacy in the fight against acute brain injury, investigation of long-term recovery could further reflect the advantages of inflammation cascade-directed therapy. Initially, it was essential to establish the optimal administration pattern for MPP-B@MM treatment. A comparative study examining different time points was conducted. As shown in Figure 4A, MCAO/R mice were treated according to four distinct administration patterns: a (MPP-B@MM at day 0), b (MPP-B@MM at day 0 and day 7), c (MPP-B@MM at day 0 and day 3), and d (MPP-B@MM at day 0, day 3, and day 7). The survival rates and performance on rotarods were displayed in Figure 4B-D. A majority of mice in group a and group b did not survive, whereas the survival rates of mice in groups c and d were significantly improved. It was indicated that injection at both day 0 and day 3 is important. Further, there was no significant difference between the results in group c and group d, which indicated that additional injection at day 7 has limited contribution to brain recovery. We hypothesize that this phenomenon may be associated with the time-dependent characteristics of stroke injury progression. Administration during the acute phase (within several hours to 24 h post-stroke) could optimize the rescue of dying neurons, aligning with the clinical principle that “time is brain” for acute treatment interventions 64. In the early subacute stage (approximately 3 days post-stroke), endogenous repair mechanisms including neurotrophic factor secretion, neurogenesis, and angiogenesis are activated 65. During this period, the administration of exogenous BDNF plasmids (MPP-B@MM) becomes crucial for facilitating the transition from acute phase treatment to neurological function recovery in clinical practice. Hence, in subsequent long-term evaluation experiments, observations were conducted with the administration of MPP-B@MM at day 0 and day 3.

### MPP-B@MM enhances the expression of BDNF in the brain and rehabilitates motor function in mice

After model establishment, MCAO/R mice were administered with different formulations at day 0 and day 3. A series of characterizations of long-term management of ischemia stroke, including behavioral tests, weight recording, and blood flow analysis were conducted over a period of three weeks (Figure 4E). Firstly, brain blood flow imaging of MCAO/R mice in different groups indicated the MPP-B@MM could facilitate recovery in infarct area (Figure 4F). The group of MCAO/R mice with Saline or BDNF plasmids alone could not even survive beyond day 5 at post-stroke. The ratio analysis of right versus left section of blood flow as shown in Figure 4G supported the statement. It was worth noting that the treatments of MPP@MM and MPP-B@MM showed similar levels of therapeutic effects at acute phase, probably due to the ROS removal of mPDA. However, MPP-B@MM showed significantly enhanced efficacy compared with MPP@MM at a later stage, which could be attributed to the effects of BDNF. In addition, the mice treated with Saline or BDNF plasmids did not receive effective treatment during the acute phase. As a result, they suffered severe brain damage and exhibited a significant weight decline. All of these mice died by the fourth day. At day 21, MCAO/R mice treated with MPP-B@MM showed higher body weight, distinctly improved modified Neurological Severity Score (mNSS) scoring and survival rate (Figure 4H-J).

To verify the advantages of acidosis-responsive drug release at infarct area, a rotarod test which reflects the neurological functions to maintain balance was carried out in the groups of responsive nanosystem (MPP-B@MM) and non-responsive BDNF-loaded nanosystem (MP-B@MM). During the first and the second week at post-stroke, no significant difference in latency to fall and rotation speed was observed between each group (Figure 4K-L). However, a notable increase of both indexes could be found at the third week. Meanwhile, we also evaluated the recovery and the motor functions of mice by adhesive removal test (Figure 4M-N). Although there was no significant difference between each group in the time to remove the sticker, the MPP-B@MM treatment was able to obviously reduce the latency to the first paper touch. The results of Elisa assay showed that the expression of BDNF in the brains of mice treated with MPP-B@MM was increased by 118% compared to MP-B@MM at day 21 (Figure 4O). The evaluation of BDNF levels in the groups of Saline, MP-B@MM and MPP-B@MM clearly demonstrated the benefits of the acidosis-responsive motif.

### *In vivo* mechanism exploration of brain pH changes after ischemia-reperfusion injury by RNA sequencing

**Figure 5:**
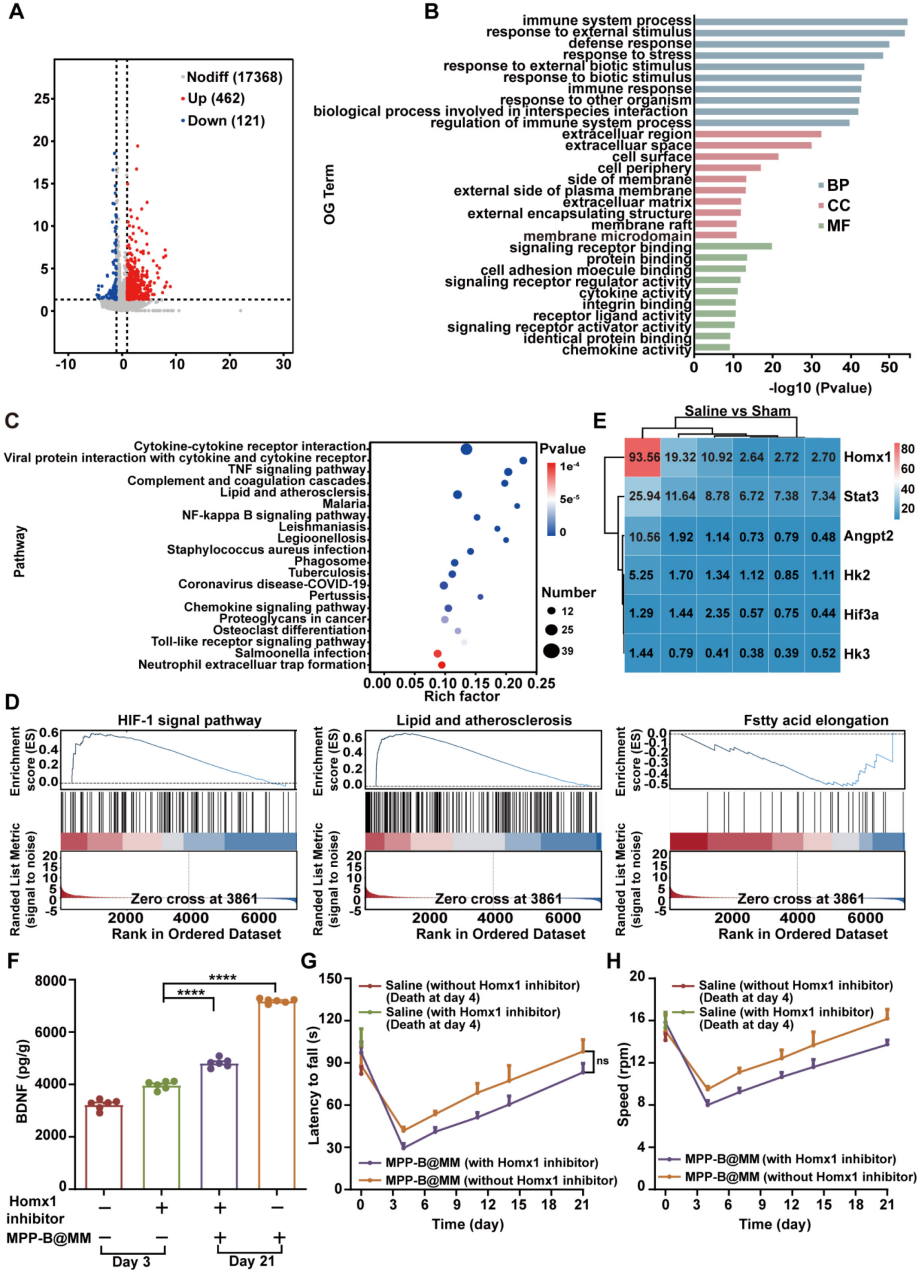
***In vivo* mechanism exploration of brain pH changes after ischemia-reperfusion injury by RNA sequencing. (A)** Sham vs Saline volcano map of differentially expressed genes. **(B)** The differential expression of 583 genes in Sham vs Saline was analyzed by GO enrichment analysis. **(C)** KEGG pathway enrichment analysis for examining the changes in signaling pathways of Sham vs Saline. **(D)** GSEA of the HIF-1, lipid and atherosclerosis, and fatty acid elongation signaling pathway as well as the lactic acid metabolic pathway. **(E)** The heatmap of typical genes positively correlated with acidity. **(F)** Expression of BDNF in the brain of mice treated with MPP-B@MM and Homx1 inhibitor for 21 days (*n* = 6). **(G)** Latency to fall of mice on the rotarod test treated with MPP-B@MM and Homx1 inhibitor for 21 days (*n* = 6). **(H)** Speed of mice on the rotarod test treated with MPP-B@MM and Homx1 inhibitor for 21 days (*n* = 6). The data are presented as means ± SEM. ****p < 0.0001 via one-way ANOVA with Dunnett's multiple comparisons test.

Acidosis is one of the key pathological processes in post-stroke inflammation cascade, accelerating the ischemia injury. To conduct a more comprehensive investigation of ischemic acidosis, brain sections from MCAO/R mice and the Sham group were collected for transcriptome analysis. Gene enrichment analysis of the brain samples based on Kyoto Encyclopedia of Genes and Genomes (KEGG) was shown in Figure 5A-E, giving the top 30 Gene Ontology (GO) terms and top 20 pathway enrichments of the two selected groups for comparison. Compared to the Sham group, obvious immune response in the ischemic brain was confirmed by a number of immune system-related activities, including immune system processes, response to external stimulus, and defense response (Figure 5B). Typical inflammatory pathways such as cytokine-cytokine receptor interaction, TNF signaling pathway, and NF-κB signaling pathway further indicated an activated inflammatory cascade in the ischemia brain (Figure 5C). Interestingly, upstream pathways related to lactic acid accumulation, including HIF-1 signaling pathway, lipid and atherosclerosis, and fatty acid elongation in the MCAO/R group showed a significant difference with that in the Sham group (Figure 5D). Among all, further gene analysis revealed that Homx1 was the crucial target for acidosis-responsive drug release and neurofunctional recovery (Figure 5E). Meanwhile, western blotting experiments confirmed that expression of Hmox1 in the injured hemispheres of mice significantly increased following MCAO/R (Figure S24). Pharmacological validation by using Hmox1 inhibitor was conducted in the MCAO/R mice groups with or without MPP-B@MM. It was found that the BDNF level was lower in the group treated with Hmox1 inhibitor, suggesting that high BDNF expression could be achieved at acidified brain region (Figure 5F). Further behavior observations at a period of three weeks after stroke also confirmed the importance of acidosis-responsive drug release. While the MPP-B@MM treatment increased the latency to fall and the speed of running mice on rotarods, administration of Hmox1 inhibitor significantly undermined the therapeutic efficacy (Figure 5G-H).

### Assessment of toxicity and immunogenicity of macrophage membrane

**Figure 6:**
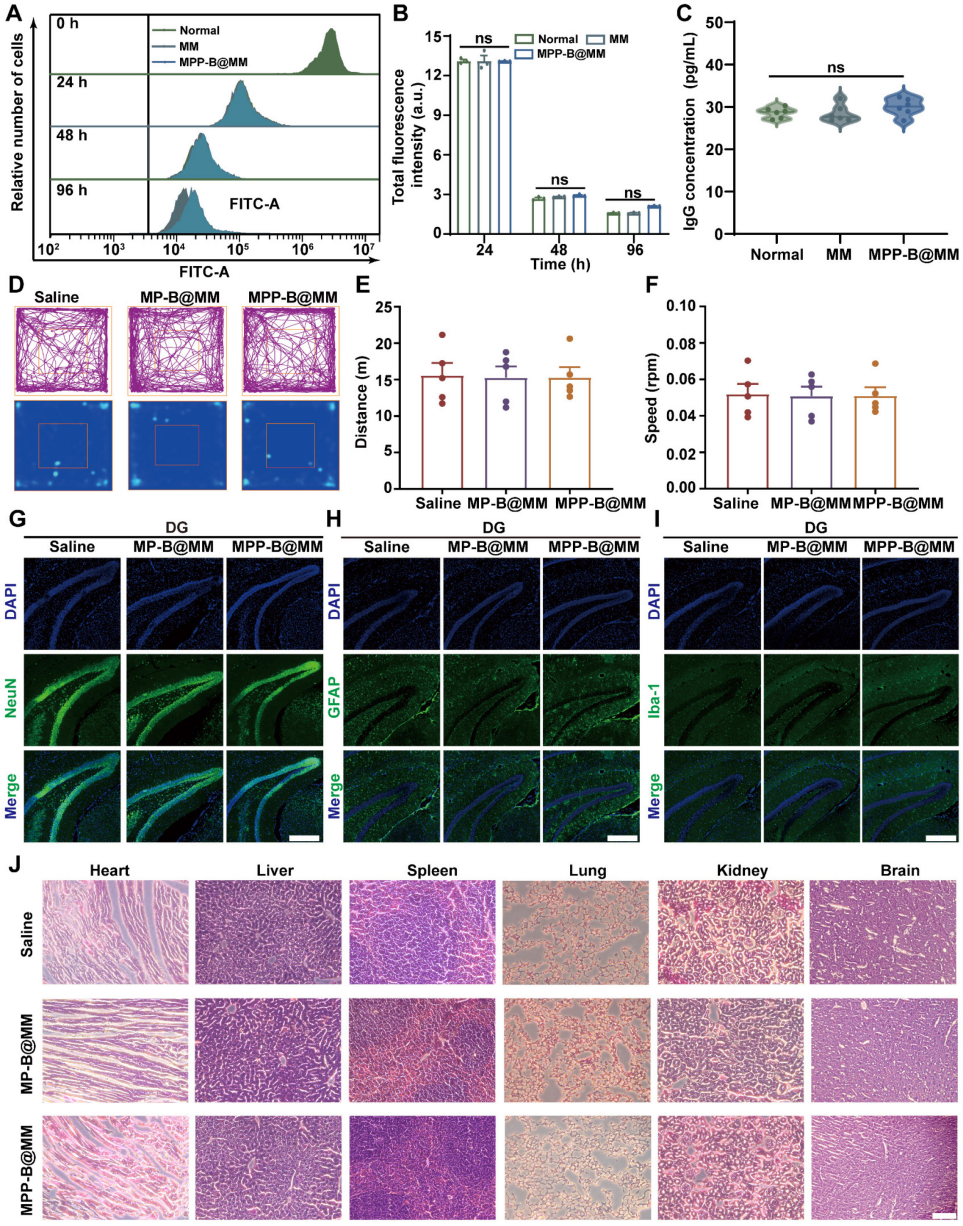
***In vivo* biosafety study of the nanosystem treatment. (A)** Jurkat, Clone E6-1 cells were labeled with CFSE and then assessed for their ability to proliferate when stimulated with MM, MPP-B@MM for 0 h, 24 h, 48 h, and 96 h. **(B)** Quantitative analysis of CFSE fluorescence intensity in A (*n* = 3). **(C)** The alterations in serum IgG levels of mice following 21 days of treatment across various groups (*n* = 6). **(D)** Typical motion route during an open-field test. **(E)** The total distance and **(F)** the center distance of mice after different treatments (*n* = 5). **(G)** Immunohistochemical staining of neurons positive cells, **(H)** astrocyte positive cells and **(I)** microglia positive cells in the dentate gyrus of mice. Scale bar, 100 μm. **(J)** H&E staining of major organs and brain after different treatments. Scale bar, 100 μm. The data are presented as means ± SEM.

Besides, Jurkat and CloneE6-1 cells were utilized to investigate the immunogenicity and toxicity of macrophage membranes. After co-incubation with Jurkat, CloneE6-1 cells for 96 h, the effects of MM and MM-BB@MM on their proliferative capacity were assessed using flow cytometry (Figure 6A-B). The results showed that the macrophage membrane did not exhibit cytotoxicity nor adversely affect T cell growth. Furthermore, at day 21 post-administration, levels of IgG, CD44, and CD62L in peripheral serum were also evaluated. There was no statistically significant difference observed among the groups when compared to the normal group (Figure 6C and S25). These findings suggested that exogenous macrophage membranes do not elicit substantial activation of the host immune system and are unlikely to provoke typical immune cell activation and migration associated with immune rejection. In addition, hemolysis experiments suggested that the bionic system has good biological safety (Figure S26).

### *In vivo* biosafety and biocompatibility evaluation

At first, an open-field test that reflects locomotor activity and anxiety-like behavior of the mice was performed in groups with different treatments. After 24 h of injection, similar running routes and heatmaps were recorded when comparing the groups of Saline and MPP-B@MM (Figure 6D). Negligible difference in running distance and speed of mice in the open-field test box was observed between each group (Figure 6E-F). Fluorescence immunostaining of different cell types in the region dentate gyrus (DG) of the hippocampus from the mice brain slices were shown in Figure 6G-I. The biomarkers, including NeuN, GFAP, and Iba-1 for neurons, astrocytes, and microglia, respectively, were captured and the results indicated no cell loss after the treatments. This is also verified at the regions of cortex and CA3 (Figure S27). Blood chemical analysis of blood urea nitrogen (BUN), alkaline phosphatase (ALP), alanine aminotransferase (ALT) and aspartate aminotransferase (AST) indicated no discernable hematological toxicity (Figure S28). At 24 h post-treatment, hematoxylin-eosin staining of brain and major organs, including heart, liver, spleen, lung, and kidney of the mice with different treatments were conducted to evaluate the potential organ toxicity. Limited histochemical changes of organ slices could be found between the groups of Saline and MPP-B@MM treatment (Figure 6J).

## Discussion

Dynamic inflammatory processes and uneven distribution of pathological changes during the post-stroke period remarkably hinder the therapeutic efficacy of anti-stroke medications. The ischemic parenchyma, which is the core region in the stroke brain, becomes acidified due to insufficient blood supply and lactic acid accumulation.

It is imperative to rescue these brain regions with pathological acidosis, which serve as a biomarker for neurological impairment in the long-term management of stroke. Further, a burst of inflammatory factors, including ROS also causes severe cellular damage, apoptosis, and irreversible cell death in the surrounding region. Thus, compared with conventional medication for brain recovery, a smart and on-demand drug administration strategy is more desirable.

To overcome the dynamic post-stroke inflammation, a biomimetic and acidosis-responsive polydopamine nanosystem is established for cascade-directed therapy. The nanosystem was fabricated by synthesis of nature-inspired mPDA and incorporation of an acidosis-responsive motif for BDNF delivery, followed by macrophage membrane coating. Here, the introduction of polydopamine has at least three discernable advantages. Firstly, the intrinsic nature of radical scavenging of polydopamine enabled removal of excessive ROS at the acute stage, and the design of mesoporous nanostructure significantly improved the scavenging efficiency due to increased specific surface area. Secondly, the mPDA nanosystem simultaneously provided sufficient room for BDNF plasmids loading and could protect the plasmids from degradation before reaching the lesion. It should be noted that the drug loading has a limited impact on the ROS removing performance. Thirdly, the unique chemistry of polydopamine allowed the catechol/boronate complexation for plasmids/PEI conjugation, which is the key to acidosis-responsive drug release. Moreover, the biomimetic strategy for surface functionalization by macrophage membrane coating endowed the nanosystem with the capability of active targeting to inflammatory brain regions. After a series of physicochemical characterizations, the custom-designed nanosystem MPP-B@MM was qualified to be a promising candidate for inflammation cascade-directed therapy in the fight against stroke.

Moreover, *in vitro* cellular study revealed the potential of brain targeting and protective effects and uncovered the mechanisms behind. An endothelial transwell model was first used to confirm the enhanced BBB transportation by biomimetic modification of macrophage membrane. Unlike passive transportation or ligand/peptide-enabled BBB penetration, the membrane-camouflaged strategy endowed the nanosystem with the BBB crossing ability via multiple pathways of which were inherited from the macrophages. Three typical factors, including ICAM-1, P-selectin, and AMPK were all proven to participate in facilitating endothelial monolayer penetration. Then, efficient ROS scavenging of MPP-B@MM nanosystem could protect the brain cells, neurons in particular, from acute injury. It was indicated that ROS scavenging by the nanosystem could rescue the cells through alleviating mitochondrial damage. Once the activity of neuron mitochondria was improved, oxidative stress and calcium overload recovered to normal and led to higher neuron survival. In addition, autophagic activity of neuron cells which remained at an elevated level was largely relieved. Through these protective pathways, the potential injury induced by deprived oxygen and glucose was fortunately decreased by MPP-B@MM nanosystem.

Further* in vivo* investigation provides more convincing evidence of the therapeutic and biosafety advantages of MPP-B@MM nanosystem in long-term management of ischemia stroke. At first, the nanosystem could accumulate at the ischemia region rather than the whole brain due to the biomimetic membrane-camouflaged strategy. Increased targeting delivery prominently reduced the acute injury at the initial stage of the brain inflammation cascade. A majority of indicators, including infarct area, neurological score, and brain water content of the stroke mice were significantly reversed. These changes were also in companied by continuous inflammation mediation and less neuron loss. Moreover, with the drug administration at day 0 and day 3, long-term recovery at the progressive stage of the cascade was achieved. Balanced blood flow between the ischemia and the normal sides, recovered weight growth, improved behavior score, and enhanced long-term survival rate proved the therapeutic performance against chronic injury of stroke. This raises the question of why higher efficacy could be obtained with MPP-B@MM treatment. We evaluated the BDNF expression at day 21 after stroke of MPP-B@MM group and found significant increment. However, the non-responsive treatment could hardly complete the mission of long-term management, indicating the importance of the acidosis-responsive motif. To gain a deeper understanding of acidosis at ischemic condition, transcriptome analysis was conducted and clearly indicated the activated inflammation-related and acidification-related pathways. Among all, Homx1 was proposed for the first time as the dominating target for acidosis-responsive drug release. As far as we know, no direct relationship has been established between the upregulation of Homx1 expression and the release of the BDNF plasmid at this time. However, through experimental investigations and prior reports, we propose a potential mechanism for their interaction. We hypothesize that this may be associated with pH changes within the stroke microenvironment. Hmox1 is a crucial enzyme primarily responsible for catalyzing the degradation of heme. The reaction yields bilirubin and divalent iron ions (Fe²⁺) as significant products, and their increased levels contribute to elevated concentrations of Fe²⁺ in the brain. Homx1 facilitates the generation of protons (H^+^) through Fe²⁺ production, which drives the Fenton reaction; this process results in alterations to both higher and lower pH levels, thereby enhancing drug release. Biosafety and biocompatibility of the treatment are crucial factors for further translational study. Characterized by behavioral observations, blood chemical analysis, immunofluorescence and histochemical staining of brain sections and major organs, our biomimetic nanosystem exhibited satisfactory biosafety profiles.

In summary, a biomimetic polydopamine nanosystem for inflammation cascade-directed therapy was developed in the fight against ischemia stroke. Taking advantage of polydopamine and mesoporous nanostructure, our nanosystem featured ischemic region targeting and efficient ROS scavenging ability. Along with the inflammation progression, the acidosis-responsive motif in the nanosystem serves as an on-demand therapeutic method, spatiotemporally increasing neurotrophic factor expression at the stroke lesion, contributing to the brain remodeling. Combined with excellent biosafety and biocompatibility, this nanosystem has displayed an effective and safe strategy for long-term management of chronic ischemia-induced injury.

## Supplementary Material

Supplementary figures.

## Abbreviations

·OH: hydroxyl radical
ABTS^+·^: 3-ethylbenzothiazoline-6-sulfonic acid
ALP: alkaline phosphatase
ALT: alanine aminotransferase
AMPK: adenosine-monophosphate activated protein kinase
AST: aspartate aminotransferase
BBB: blood-brain barrier
BCA: Bicinchoninic Acid Assay
BDNF: brain derived neurotrophic factor
bEnd.3: brain-derived Endothelial cells.3
BUN: blood urea nitrogen
CCA: common carotid artery
CLSM: Confocal Laser Scanning Microscopy
DA·HCl: dopamine hydrochloride
DAPI: 4',6-Diamidino-2-phenylindole
DHE: dihydroethidium
DMEM: dulbecco's modified Eagle's medium
DPPH·: 1,1-diphenyl-2-picryl-hydrazyl
EDC: 1-(3-Dimethylaminopropyl)-3-ethylcarbodiimide
Elisa: Enzyme-linked immunosorbent assay
FBS: fetal bovine serum
GO: Gene Ontology
H_2_O_2_: hydrogen peroxide
ICG: indocyanine green
ICAM-1: intercellular adhesion molecule-1
IL-1β: Interleukin-1β
KEGG: Kyoto Encyclopedia of Genes and Genomes
MCA: middle cerebral artery
MCAO/R: middle cerebral artery occlusion/reperfusion
MM: macrophage membrane
mPDA: mesoporous polydopamine
NHS: N-Hydroxy succinimide
O^2·-^: super oxide anion radical
OGD/R: oxygen-glucose deprivation/reoxygenation
OGD: oxygen-glucose deprivation
OTC: optimal cutting temperature compound
PBA: 4-carboxyphenylboronic acid
PBS: phosphate-buffered saline
PEI: Polyetherimide
ROS: reactive oxygen species
SDS-PAGE: sodium dodecyl sulfate -polyacrylamide gel electrophoresis
SEM: scanning electron microscopy
TEER: trans-endothelial electrical resistance
TEM: transmission electron microscopy
TMB: 3, 3', 5, 5'-Tetramethylbenzidine
TNF-α: Tumour necrosis factor-α
TTC: 2, 3, 5-triphenyltetrazolium chloride
TUNEL: Terminal deoxynucleotidyl transferase-mediated dUTP nick end labeling
