# Supplementary material for: Inflammation Cascade-Directed Therapy by Biomimetic Polydopamine Nanosystem for Long-Term Management of Ischemic Stroke
Source: Theranostics. 2026 Jan 1;16(1):77–98. doi: 10.7150/thno.118196 (PMC12665104; doi:10.7150/thno.118196)
Supplement: Supplementary file 1 — Supplementary figures. [file thnov16p0077s1.pdf]

Inflammation cascade-directed therapy by biomimetic polydopamine nanosystem for long-term management of ischemic stroke

Yuyi Zheng[†,1], Xiaojie Chen[†,1], Qi Zhang[1], Ziwei Xu[1], Yixuan Gan[1], Jing Zhou[1], Wenlu Li[1], Yi Wang[1], Zhong Chen[1,*], Di Wu[1,*]

1. Zhejiang Collaborative Innovation Center for the Brain Diseases with Integrative Medicine, Zhejiang Key Laboratory of Neuropsychopharmacology, School of Pharmaceutical Sciences and School of Basic Medical Sciences, The Fifth Affiliated Hospital (Huzhou Central Hospital), Zhejiang Chinese Medical University, Hangzhou, China.

†These authors contributed equally to this work.
Corresponding author: Di Wu, wudichem@zju.edu.cn; Zhong Chen, chenzhong@zju.edu.cn.

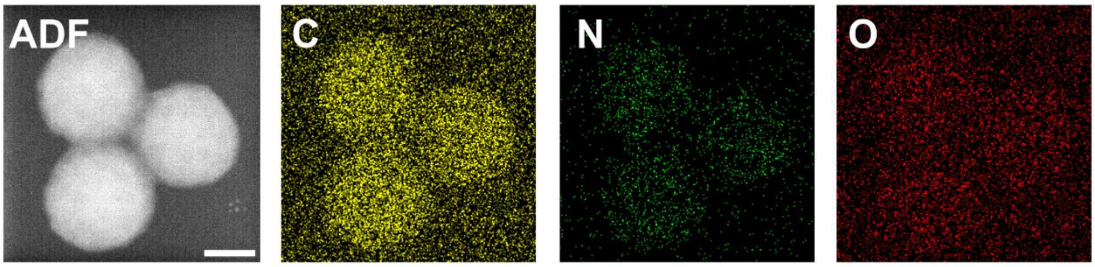

Figure S1. TEM mapping of the elements for mPDA. Scale bar, 50 nm.

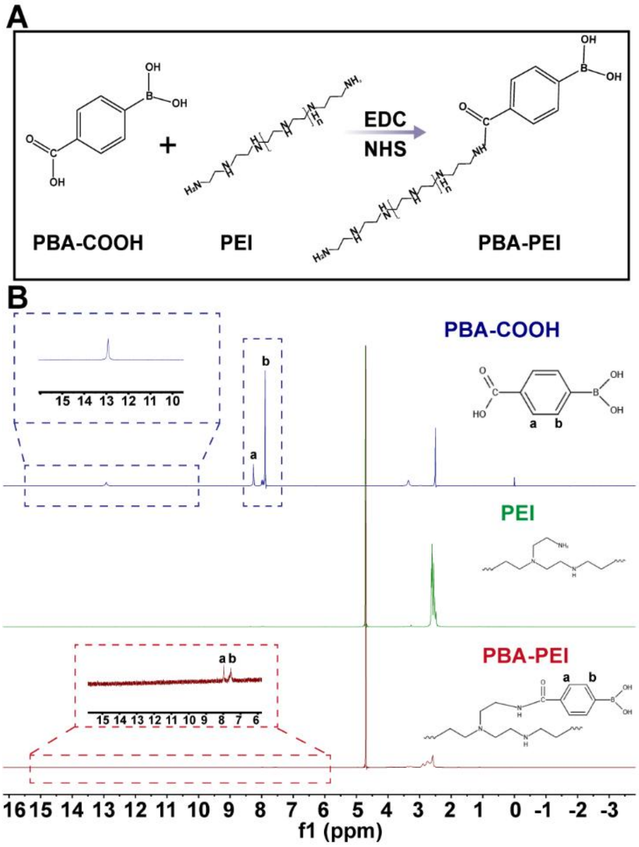

Figure S2. (A) Synthesis process of PBA-PEI. (B) ^{1}H NMR spectrum of PBA-COOH, PEI, and PBA-PEI.

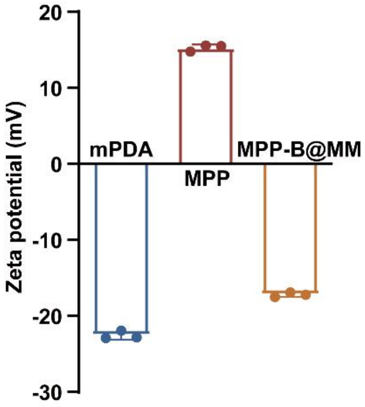

Figure S3. Zeta potentials of mPDA, MPP, and MPP-B@MM (n = 3). The data are presented as means ± SEM.

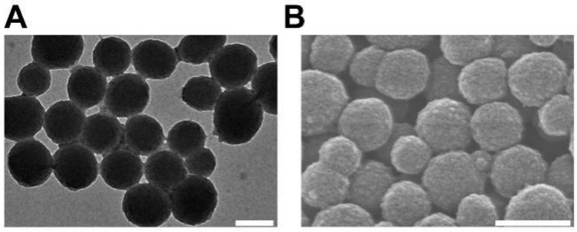

Figure S4. (A) TEM, and (B) SEM images of sPDA. Scale bar, 200 nm.

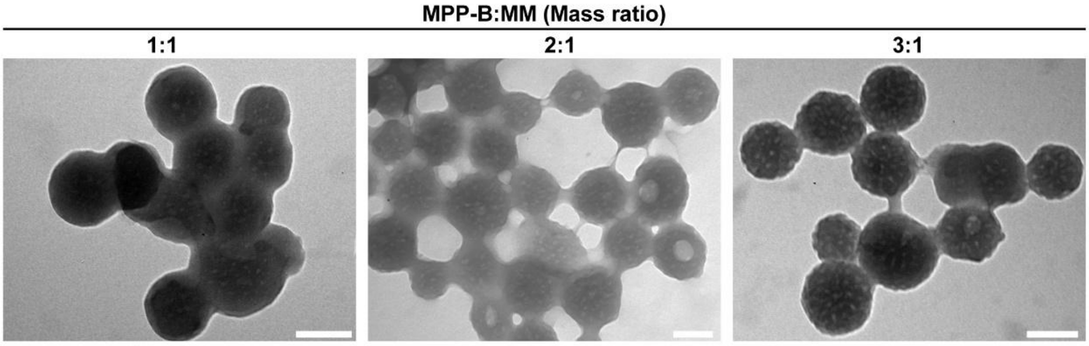

Figure S5. TEM images of MPP-B@MM at different mass ratios of MPP-B@MM. Scale bar, 100 nm.

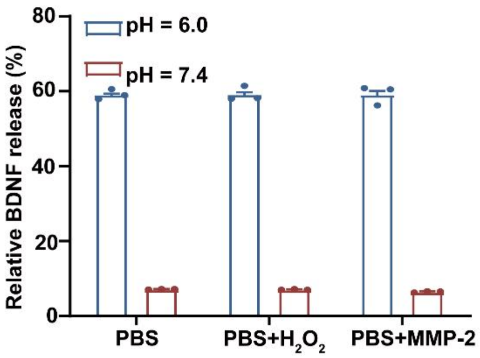

Figure S6. Drug release results of the nanosystem in PBS, and in the presence of H_2O_2 (100 μM) or matrix metallopeptidase-2 (MMP-2, 100 ng/mL) (n = 3). The data are presented as means ± SEM.

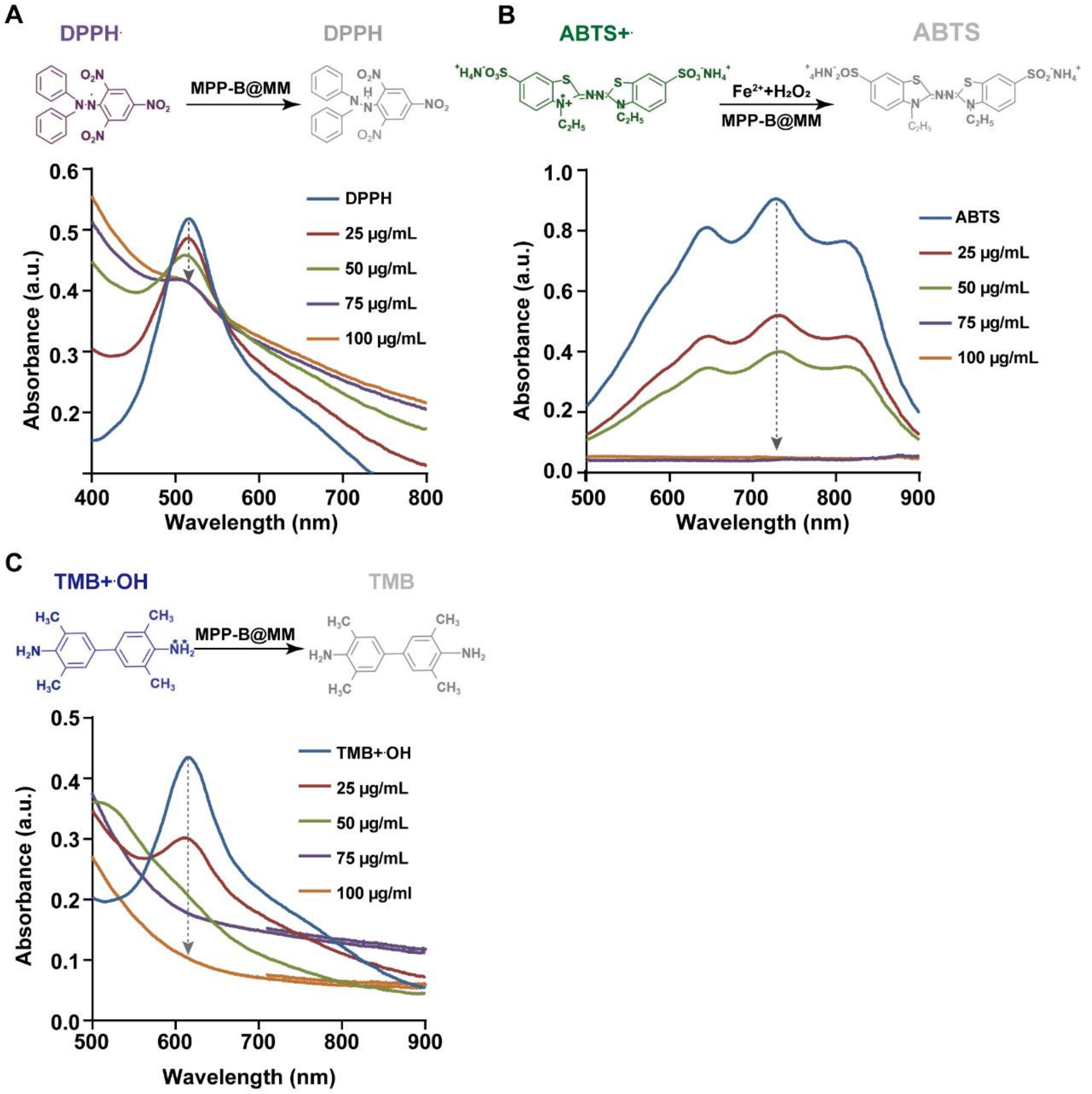

Figure S7. UV-vis absorbance spectra of (A) DPPH·, (B) $ABTS^{+\cdot}$, and (C) ·OH radical after incubation with different concentration gradients of MPP-B@MM.

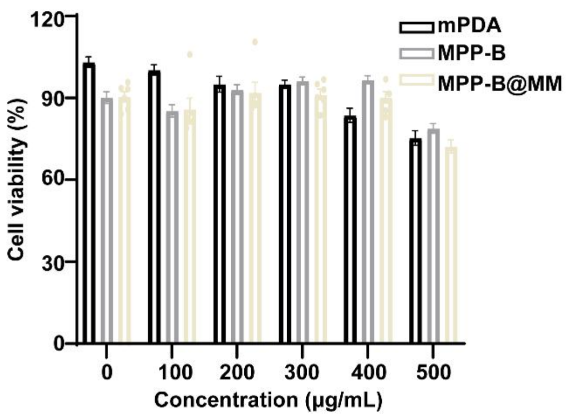

Figure S8. Cytotoxicity evaluation of SH-SY5Y at different concentrations of mPDA,

MPP-B and MPP-B@MM (n = 6). The data are presented as means ± SEM.

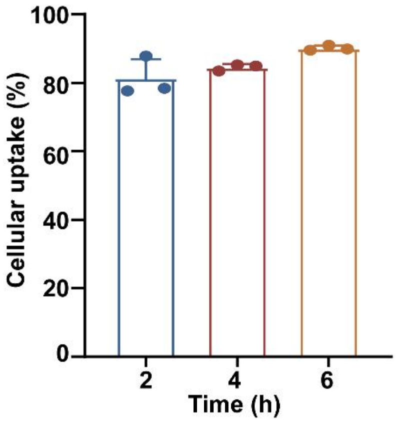

Figure S9. Flow cytometry analysis of bEnd.3 cells incubated with MPP-B@MM for different (2, 4, and 6 h) (n =). The data are presented as means ± SEM.

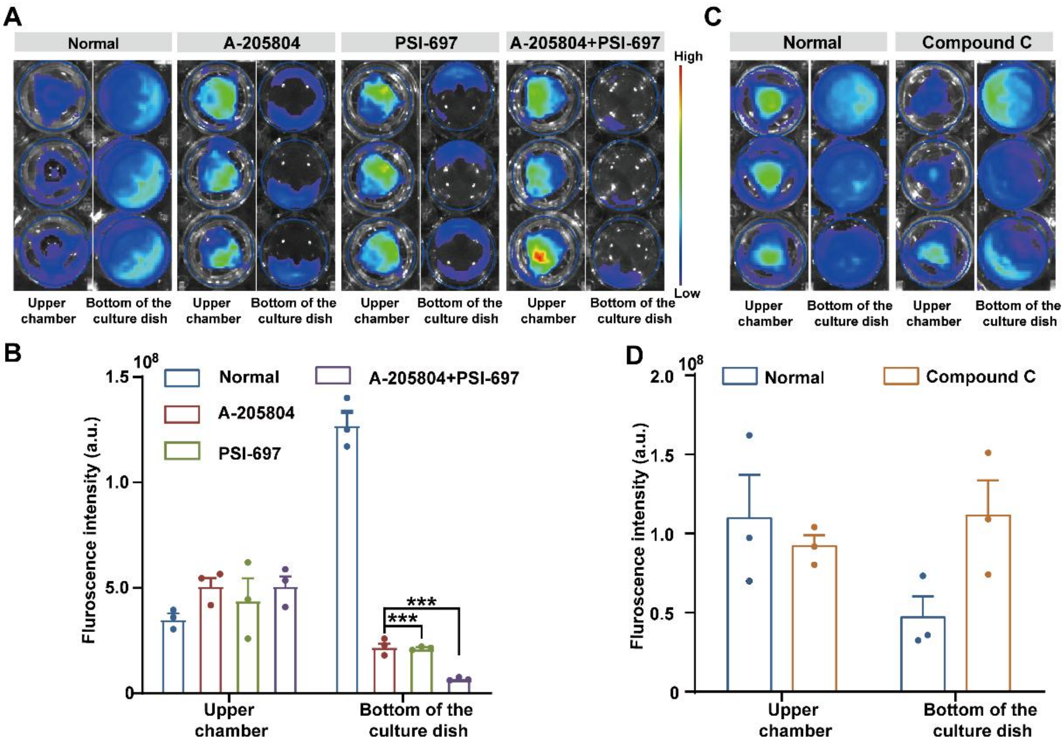

Figure S10. (A) Fluorescence images of the lower and upper chamber after incubation of bEnd.3 with A-205804, and PSI-697. (B) Average relative fluorescence was estimated from images in (A) (n = 3). (C) Fluorescence images of the lower and upper chamber after incubation of bEnd.3 with Compound C. (D) The relative fluorescence intensity was estimated from images in (C) (n = 3). The data are presented as means ± SEM. ***p < 0.001 via one-way ANOVA with Dunnett's

multiple comparisons test.

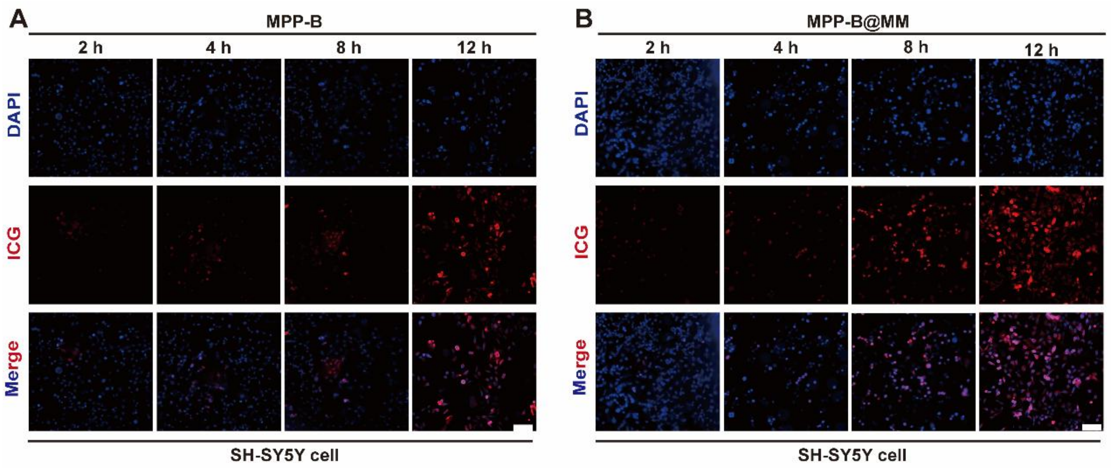

Figure S11. Fluorescence images of (A) MPP-B, and (B) MPP-B@MM after incubated with SH-SY5Y cells for 2, 4, 8 and 12 h. Scale bar, 100 µm.

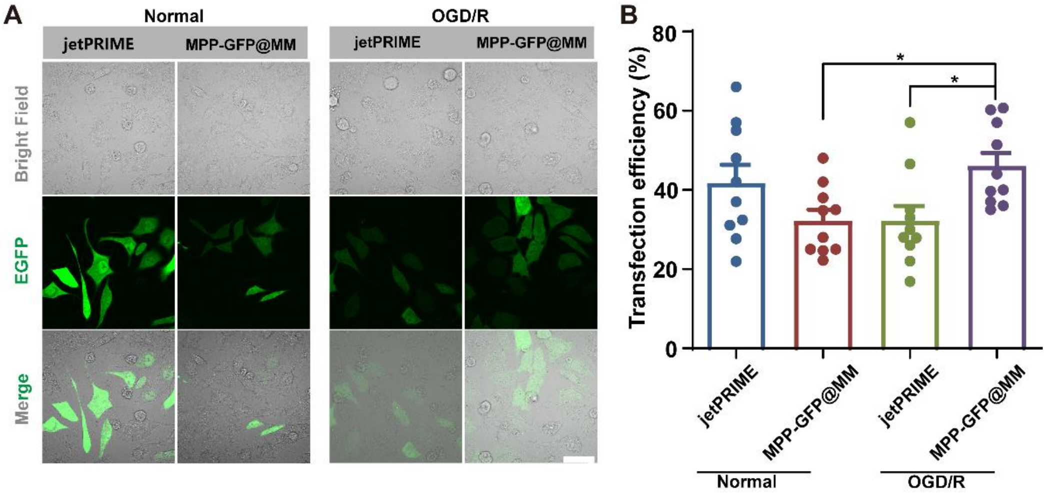

Figure S12. (A) Fluorescence images of green fluorescent protein expression by MPP-GFP@MM on SH-SY5Y cells. Scale bar, 50 µm. (B) Transfection efficiency was estimated from image in (A) (n = 10). The data are presented as means ± SEM. *$p < 0.05$ via one-way ANOVA with Dunnett's multiple comparisons test.

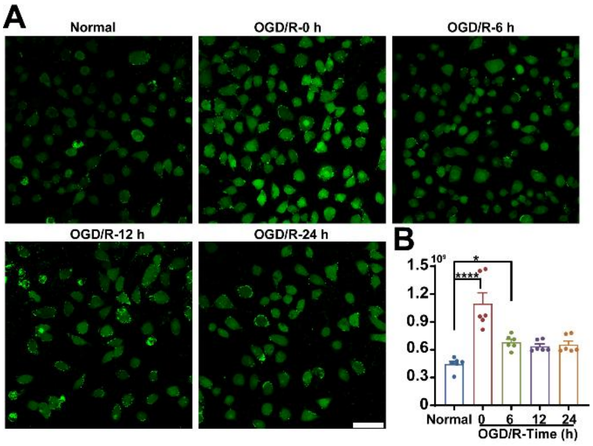

Figure S13. (A) Representative images of SH-SY5Y cells stained with pHrodo fluorescence probe. Scale bar, 50 μm. (B) Average relative fluorescence was estimated from images in G (n = 6). The data are presented as means ± SEM. *$p < 0.05$, ****$p < 0.0001$ via one-way ANOVA with Dunnett's multiple comparisons test.

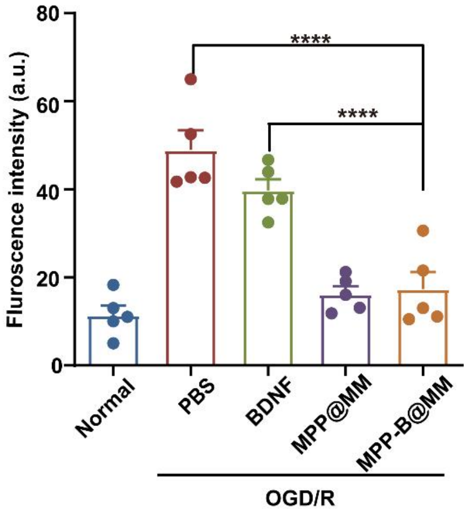

Figure S14. Relative fluorescence intensity was estimated from images in Figure 2G (n = 5). The data are presented as means ± SEM. ****$p < 0.0001$ via one-way ANOVA with Dunnett's multiple comparisons test.

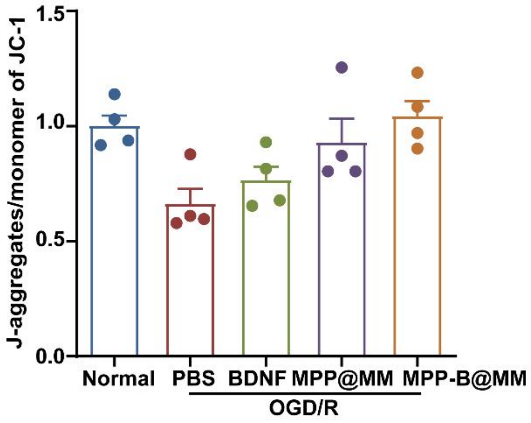

Figure S15. JC-1 aggregates/JC-1 monomer fluorescence ratio was analyzed to indicate mitochondrial membrane potential (n = 4). The data are presented as means ± SEM.

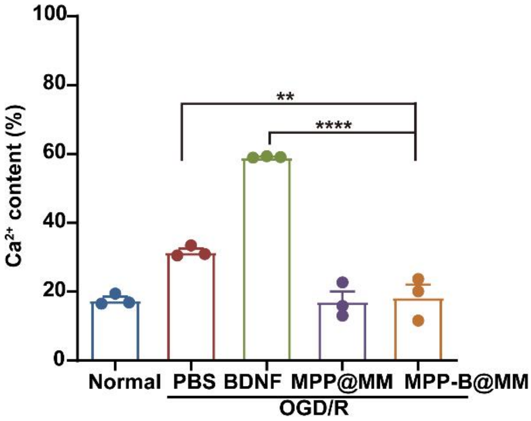

Figure S16. Quantitative analysis of intracellular Ca^{2+} levels (n = *3*). The data are presented as means ± SEM. **p < 0.01, ****p < 0.0001 via one-way ANOVA with Dunnett's multiple comparisons test.

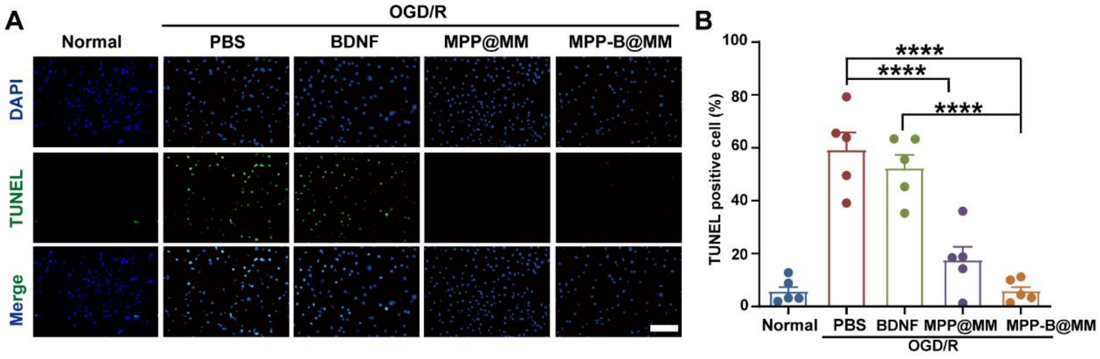

Figure S17. Representative images of TUNEL staining. Scale bar, 100 μm. TUNEL positive cell was estimated from images in Figure S17A (n = 5). The data are presented as means ± SEM. ****p < 0.0001 via one-way ANOVA with Dunnett's multiple comparisons test.

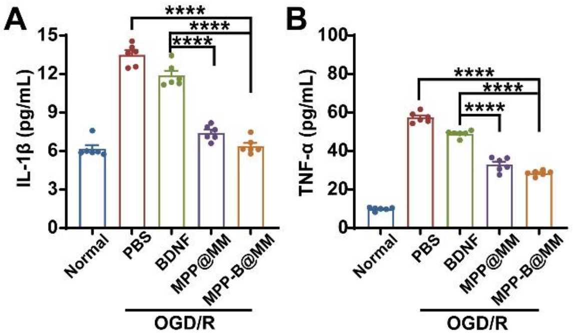

Figure S18. (A) IL-β and (B) TNF-α were detected by Elisa kit (n = 6). The data are presented as means ± SEM. ****p < 0.0001 via one-way ANOVA with Dunnett's multiple comparisons test.

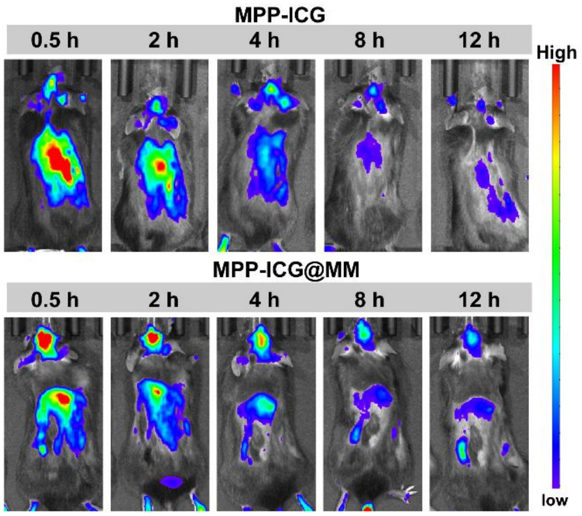

Figure S19. *In vivo* fluorescence images of MCAO/R mice following a single intravenous injection of MPP-ICG and MPP-ICG@MM.

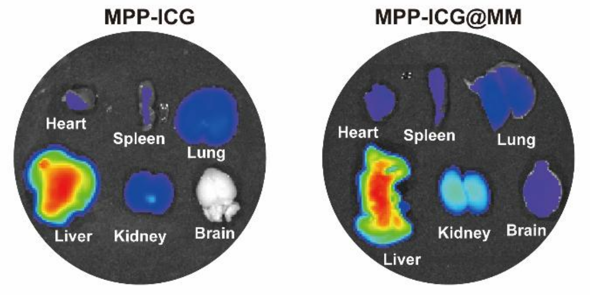

Figure S20. *Ex vivo* imaging of the major organs of the MCAO/R mice treated with MPP-ICG or MPP-ICG@MM.

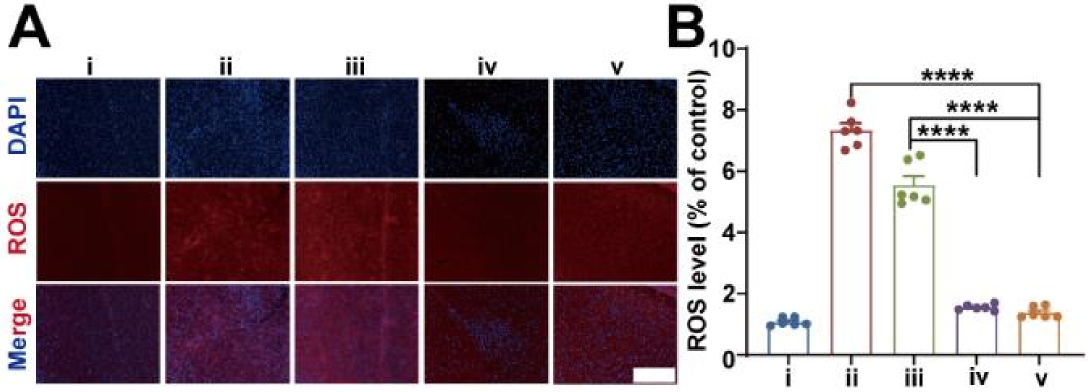

Figure S21. (A) DHE was used for brain section staining to detect the ROS levels. Scale bar, 100 μm. (B) The relative DHE fluorescence intensity was measured using Microplate Reader (n = 6). i: Sham, ii: Saline, iii: BDNF, iv: MPP@MM, v: MPP-B@MM. The data are presented as means ± SEM. ****p < 0.0001 via one-way ANOVA with Dunnett's multiple comparisons test.

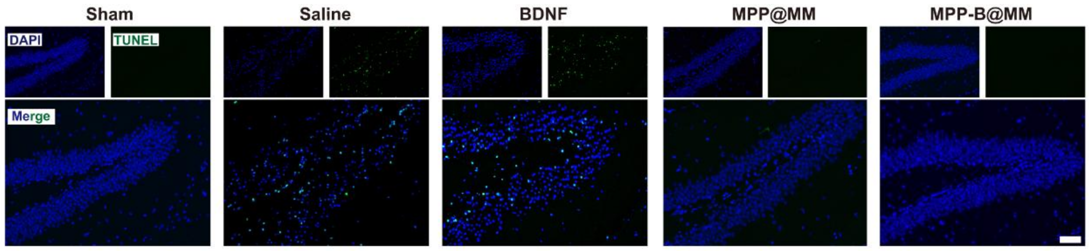

Figure S22. Representative images of TUNEL staining in the cortex region. Scale bar, 100 μm.

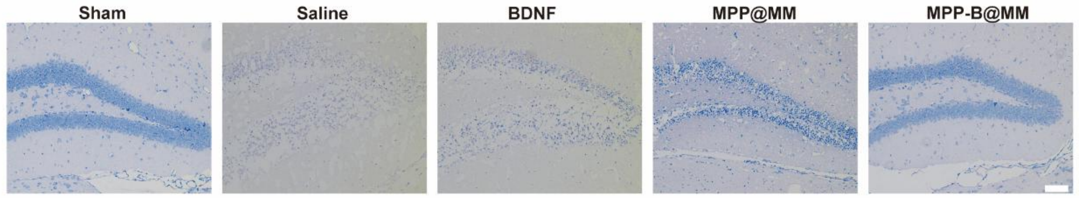

Figure S23. Representative images of Nissl staining in the hippocampal region. Scale bar, 100 μm.

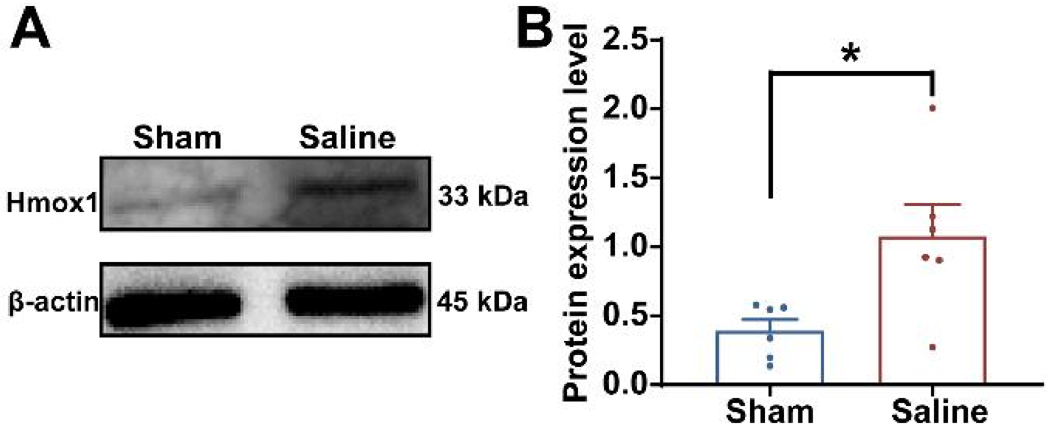

Figure S24. (A) Western blot analysis and (B) quantification of Hmox1 in infarcted hemibrain in mice (n = 6). The data are presented as means ± SEM. *p < 0.05 via one-way ANOVA with Dunnett's multiple comparisons test.

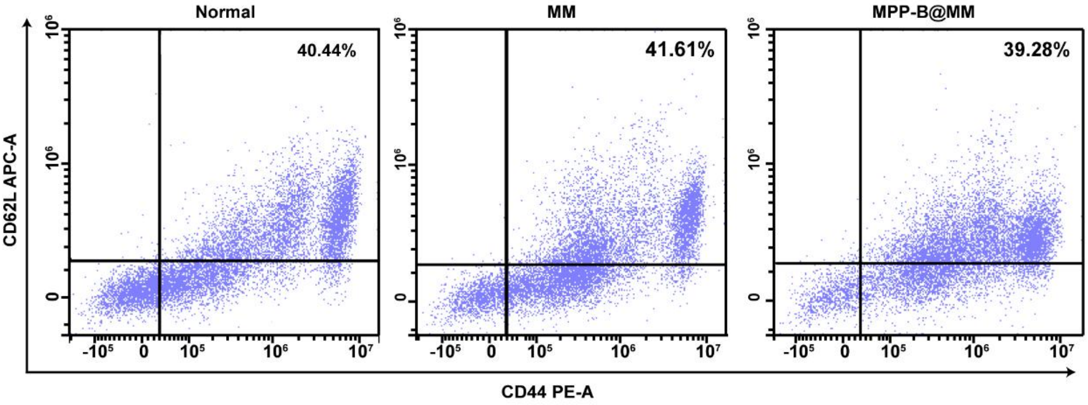

Figure S25. The alterations in serum CD62L and CD44 levels of mice treated with MM or MPP-B@MM.

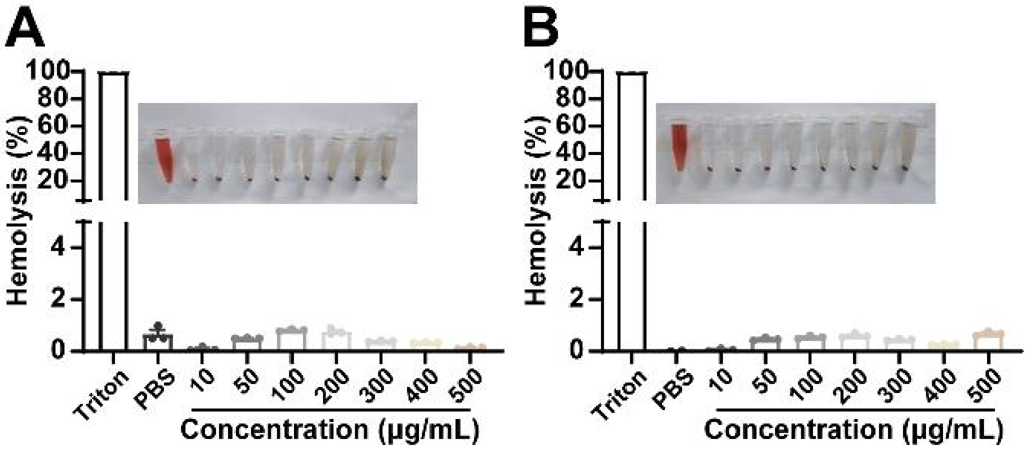

Figure S26. Hemolysis rate of MPP-B (A) and MPP-B@MM (B) (n = 3).

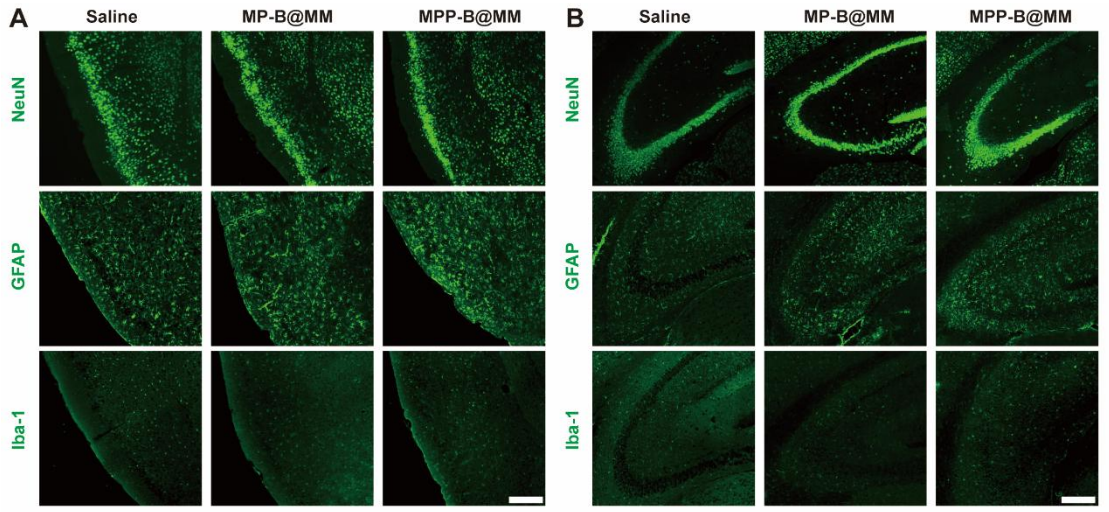

Figure S27. Immunohistochemical staining of positive cells in the (A) cortex, and (B) hippocampus region of mice. Scale bar, 100 μm.

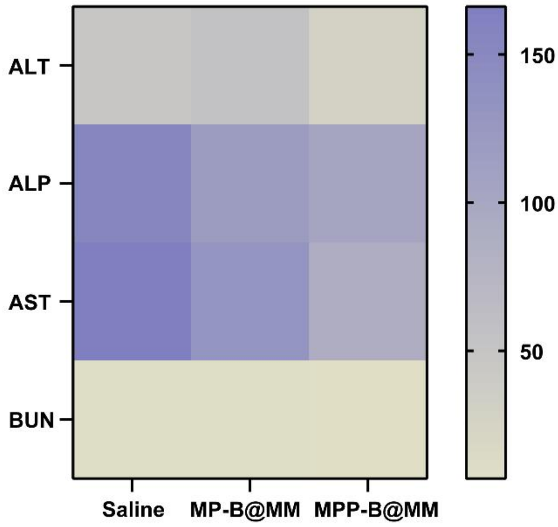

Figure S28. Hematological analysis of alanine aminotransferase (ALT), alkaline phosphatase (ALP), aspartate transaminase (AST), and blood urea nitrogen (BUN) level (n = 5). The data are presented as means ± SEM.
